# Estimating genomic coexpression networks using first-order conditional independence

**DOI:** 10.1186/gb-2004-5-12-r100

**Published:** 2004-11-30

**Authors:** Paul M Magwene, Junhyong Kim

**Affiliations:** 1 Department of Biology, University of Pennsylvania, 415 S University Avenue, Philadelphia, PA 19104, USA; 2 Current address: Department of Biology, Duke University, Durham, NC 27708, USA

## Abstract

We describe a computationally efficient statistical framework for estimating networks of coexpressed genes. This framework exploits first-order conditional independence relationships among gene-expression measurements to estimate patterns of association. We use this approach to estimate a coexpression network from microarray gene-expression measurements from *Saccharomyces cerevisiae*. We demonstrate the biological utility of this approach by showing that a large number of metabolic pathways are coherently represented in the estimated network. We describe a complementary unsupervised graph search algorithm for discovering locally distinct subgraphs of a large weighted graph. We apply this algorithm to our coexpression network model and show that subgraphs found using this approach correspond to particular biological processes or contain representatives of distinct gene families.

## Abstract 2

A computationally efficient statistical framework for estimating networks of coexpressed genes is presented that exploits first-order conditional independence relationships among gene expression measurements.

## Background

Analyses of functional genomic data such as gene-expression microarray measurements are subject to what has been called the 'curse of dimensionality'. That is, the number of variables of interest is very large (thousands to tens of thousands of genes), yet we have relatively few observations (typically tens to hundreds of samples) upon which to base our inferences and interpretations. Recognizing this, many investigators studying quantitative genomic data have focused on the use of either classical multivariate techniques for dimensionality reduction and ordination (for example, principal component analysis, singular value decomposition, metric scaling) or on various types of clustering techniques, such as hierarchical clustering [1], *k*-means clustering [2], self-organizing maps [3] and others. Clustering techniques in particular are based on the idea of assigning either variables (genes or proteins) or objects (such as sample units or treatments) to equivalence classes; the hope is that equivalence classes so generated will correspond to specific biological processes or functions. Clustering techniques have the advantage that they are readily computable and make few assumptions about the generative processes underlying the observed data. However, from a biological perspective, assigning genes or proteins to single clusters may have limitations in that a single gene can be expressed under the action of different transcriptional cascades and a single protein can participate in multiple pathways or processes. Commonly used clustering techniques tend to obscure such information, although approaches such as fuzzy clustering (for example, Höppner *et al*. [4]) can allow for multiple memberships.

An alternate mode of representation that has been applied to the study of whole-genome datasets is network models. These are typically specified in terms of a graph, *G *= {*V*,*E*}, composed of vertices (*V*; the genes or proteins of interest) and edges (*E*; either undirected or directed, representing some measure of 'interaction' between the vertices). We use the terms 'graph' and 'network' interchangeably throughout this paper. The advantage of network models over common clustering techniques is that they can represent more complex types of relationships among the variables or objects of interest. For example, in distinction to standard hierarchical clustering, in a network model any given gene can have an arbitrary number of 'neighbors' (that is *n*-ary relationships) allowing for a reasonable description of more complex inter-relationships.

While network models seem to be a natural representation tool for describing complex biological interactions, they have a number of disadvantages. Analytical frameworks for estimating networks tend to be complex, and the computation of such models can be quite hard (NP-hard in many cases [5]). Complex network models for very large datasets can be difficult to visualize; many graph layout problems are themselves NP-hard. Furthermore, because the topology of the networks can be quite complex, it is a challenge to extract or highlight the most 'interesting' features of such networks.

Two major classes of network-estimation techniques have been applied to gene-expression data. The simpler approach is based on the notion of estimating a network of interactions by defining an association threshold for the variables of interest; pairwise interactions that rise above the threshold value are considered significant and are represented by edges in the graph, interactions below this threshold are ignored. Measures of association that have been used in this context include Pearson's product-moment correlation [6] and mutual information [7]. Whereas network estimation using this approach is computationally straightforward, an important weakness of simple pairwise threshold methods is that they fail to take into account additional information about patterns of interaction that are inherent in multivariate datasets. A more principled set of approaches for estimating co-regulatory networks from gene-expression data are graphical modeling methods, which include Bayesian networks and Gaussian graphical models [8-11]. The common representation that these techniques employ is a graph theoretical framework in which the vertices of the graph represent the set of variables of interest (either observed or latent), and the edges of the graph link pairs of variables that are not conditionally independent. The graphs in such models may be either undirected (Gaussian graphical models) or directed and acyclic (Bayesian networks). The appeal of graphical modeling techniques is that they represent a distribution of interest as the product of a set of simpler distributions taking into account conditional relationships. However, accurately estimating graphical models for genomic datasets is challenging, in terms of both computational complexity and the statistical problems associated with estimating high-order conditional interactions.

We have developed an analytical framework, called a first-order conditional independence (FOCI) model, that strikes a balance between these two categories of network estimation. Like graphical modeling techniques, we exploit information about conditional independence relationships - hence our method takes into account higher-order multivariate interactions. Our method differs from standard graphical models because rather than trying to account for conditional interactions of all orders, as in Gaussian graphical models, we focus solely on first-order conditional independence relationships. One advantage of limiting our analysis to first-order conditional interactions is that in doing so we avoid some of the problems of power that we encounter if we try to estimate very high-order conditional interactions. Thus this approach, with the appropriate caveats, can be applied to datasets with moderate sample sizes. A second reason for restricting our attention to first-order conditional relationships is computational complexity. The running time required to calculate conditional correlations increases at least exponentially as the order of interactions increases. The running time for calculating first-order interactions is worst case *O*(*n*^3^). Therefore, the FOCI model is readily computable even for very large datasets.

We demonstrate the biological utility of the FOCI network estimation framework by analyzing a genomic dataset representing microarray gene-expression measurements for approximately 5,000 yeast genes. The output of this analysis is a global network representation of coexpression patterns among genes. By comparing our network model with known metabolic pathways we show that many such pathways are well represented within our genomic network. We also describe an unsupervised algorithm for highlighting potentially interesting subgraphs of coexpression networks and we show that the majority of subgraphs extracted using this approach can be shown to correspond to known biological processes, molecular functions or gene families.

## Results

We used the FOCI network model to estimate a coexpression network for 5,007 yeast open reading frames (ORFs). The data for this analysis are drawn from publicly available microarray measurements of gene expression under a variety of physiological conditions. The FOCI method assumes a linear model of association between variables and computes dependence and independence relationships for pairs of variables up to a first-order (that is, single) conditioning variable. More detailed descriptions of the data and the network estimation algorithm are provided in the Materials and methods section.

**Figure 1:**
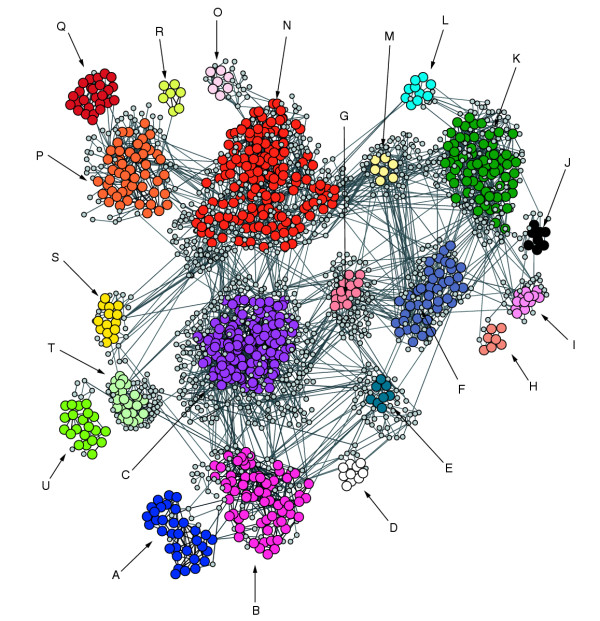
Simplification of the yeast FOCI coexpression network constructed by retaining the 4,000 strongest edges (= 1,729 vertices). The colored vertices represent a subset of the locally distinct subgraphs of the FOCI network; letters are as in Table 2, and further details can be found there. Some of the locally distinct subgraphs of Table 2 are not represented in this figure because they involve subgraphs whose edge weights are not in the top 4,000 edges.

On the basis of an edge-wise false-positive rate of 0.001 (see Materials and methods), the estimated network for the yeast expression data has 11,450 edges. It is possible for the FOCI network estimation procedure to yield disconnected subgraphs - that is, groups of genes that are related to each other but not connected to any other genes. However, the yeast coexpression network we estimated includes a single giant connected component (GCC, the largest subgraph such that there is a path between every pair of vertices) with 4,686 vertices and 11,416 edges. The next largest connected component includes only four vertices; thus the GCC represents the relationships among the majority of the genes in the genome. In Figure 1 we show a simplification of the FOCI network constructed by retaining the 4,000 strongest edges. We used this edge-thresholding procedure to provide a comprehensible two-dimensional visualization of the graph; all the results discussed below were derived from analyses of the entire GCC of the FOCI network.

**Figure 2:**
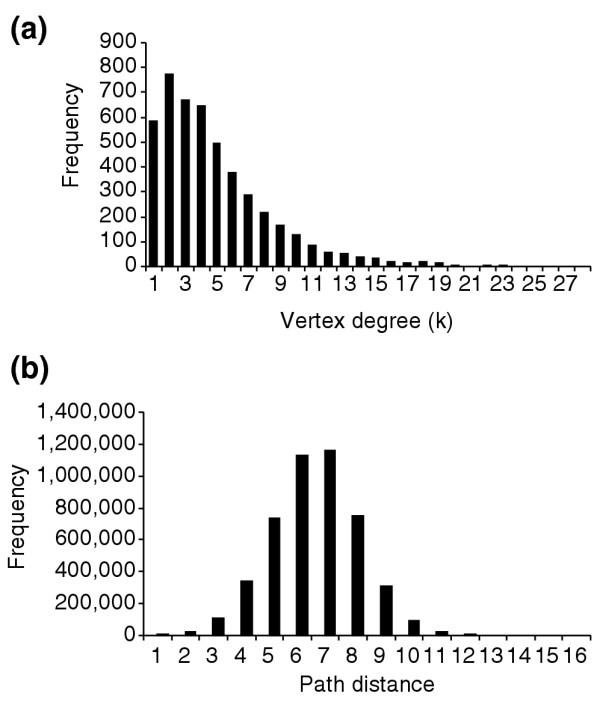
Topological properties of the yeast FOCI coexpression network. Distribution of **(a) **vertex degrees and **(b) **path lengths for the network.

The mean, median and modal values for vertex degree in the GCC are 4.87, 4 and 2 respectively. That is, each gene shows significant expression relationships to approximately five other genes on average, and the most common form of relationship is to two other genes. Most genes have five or fewer neighbors, but there is a small number of genes (349) with more than 10 neighbors in the FOCI network; the maximum degree in the graph is 28 (Figure 2a). Thus, approximately 7% of genes show significant expression relationships to a fairly large number of other genes. The connectivity of the FOCI network is not consistent with a power-law distribution (see Additional data file 1 for a log-log plot of this distribution). We estimated the distribution of path distances between pairs of genes (defined as the smallest number of graph edges separating the pair) by randomly choosing 1,000 source vertices in the GCC, and calculating the path distance from each source vertex to every other gene in the network (Figure 2b). The mean path distance is 6.46 steps, and the median is 6.0 (mode = 7). The maximum path distance is 16 steps. Therefore, in the GCC of the FOCI network, random pairs of genes are typically separated by six or seven edges.

### Coherence of the FOCI network with known metabolic pathways

To assess the biological relevance of our estimated coexpression network we compared the composition of 38 known metabolic pathways (Table 1) to our yeast coexpression FOCI network. In a biologically informative network, genes that are involved in the same pathway(s) should be represented as coherent pieces of the larger graph. That is, under the assumption that pathway interactions require co-regulation and coexpression, the genes in a given pathway should be relatively close to each other in the estimated global network.

We used a pathway query approach to examine 38 metabolic pathways relative to our FOCI network. For each pathway, we computed a quantity called the 'coherence value' that measures how well the pathway is recovered in a given network model (see Materials and methods). Of the 38 pathways tested, 19 have coherence values that are significant when compared to the distribution of random pathways of the same size (*p *< 0.05; see Materials and methods). Most of the pathways of carbohydrate and amino-acid metabolism that we examined are coherently represented in the FOCI network. Of each of the major categories of metabolic pathways listed in Table 1, only lipid metabolism and metabolism of cofactors and vitamins are not well represented in the FOCI network.

The five largest coherent pathways are glycolysis/gluconeogenesis, the TCA cycle, oxidative phosphorylation, purine metabolism and synthesis of N-glycans. Other pathways that are distinctive in our analysis include the glyoxylate cycle (6 of 12 genes in largest coherent subnetwork), valine, leucine, and isoleucine biosynthesis (10 of 15 genes), methionine metabolism (6 of 13 genes), phenylalanine, tyrosine, and tryptophan metabolism (two subnetworks each of 6 genes). Several coherent subsets of the FOCI network generated by these pathway queries are illustrated in the Additional data file 1.

### Combined analysis of core carbohydrate metabolism

In addition to being consistent with individual pathways, a useful network model should capture interactions between pathways. To explore this issue we queried the FOCI network on combined pathways and again measured its coherence. We illustrate one such combined query based on four related pathways involved in carbohydrate metabolism: glycolysis/gluconeogenesis, pyruvate metabolism, the TCA cycle and the glyoxylate cycle.

**Figure 3:**
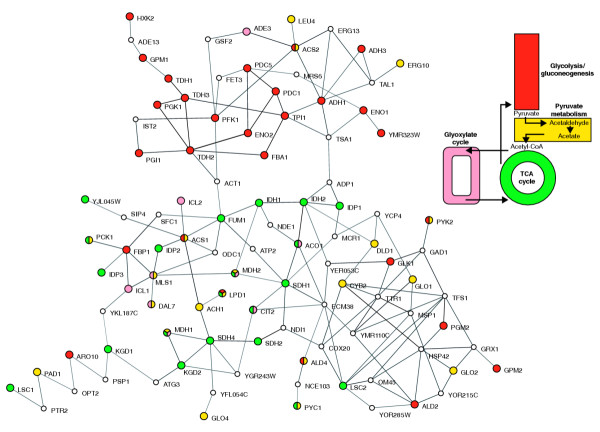
Largest connected subgraph resulting from combined query on four pathways involved in carbohydrate metabolism: glycolysis/gluconeogenesis (red); pyruvate metabolism (yellow); TCA cycle (green); and the glyoxylate cycle (pink). Genes encoding proteins involved in more than one pathway are highlighted with multiple colors. Uncolored vertices represent non-pathway genes that were recovered in the combined pathway query. See text for further details.

Figure 3 illustrates the largest subgraph extracted in this combined analysis. The combined query results in a subset of the FOCI network that is larger than the sum of the subgraphs estimated separately from individual pathways because it also admits non-query genes that are connected to multiple pathways. The nodes of the graph are colored according to their membership in each of the four pathways as defined by the Kyoto Encyclopedia of Genes and Genomes (KEGG). Many gene products are assigned to multiple pathways. This is particularly evident with respect to the glyoxylate cycle; the only genes uniquely assigned to this pathway are *ICL1 *(encoding an isocitrate lyase) and *ICL2 *(a 2-methylisocitrate lyase).

In this combined pathway query the TCA cycle, glycolysis/gluconeogenesis, and glyxoylate cycle are each represented primarily by a single two-step connected subgraph (see Materials and methods). Pyruvate metabolism on the other hand, is represented by at least two distinct subgraphs, one including {*PCK1*, *DAL7*, *MDH2*, *MLS1*, *ACS1*, *ACH1*, *LPD1*, *MDH1*} and the other including {*GLO1*, *GLO2*, *DLD1*, *CYB2*}. This second set of genes encodes enzymes that participate in a branch of the pyruvate metabolism pathway that leads to the degradation of methylglyoxal (methylglyoxal → L-lactaldehyde → L-lactate → pyruvate and methylglyoxal → (*R*)-*S*-lactoyl-glutathione → D-lactaldehyde → D-lactate → pyruvate) [12,13]. In the branch of methylglyoxal metabolism that involves *S*-lactoyl-glutathione, methyglyoxal is condensed with glutathione [12]. Interestingly, two neighboring non-query genes, *GRX1 *(a neighbor of *GLO2*) and *TTR1 *(neighbor of *CYB2*), encode proteins with glutathione transferase activity.

The position of *FBP1 *in the combined query is also interesting. The product of *FBP1 *is fructose-1,6-bisphosphatase, an enzyme that catalyzes the conversion of beta-d-fructose 1,6-bisphosphate to beta-D-fructose 6-phosphate, a reaction associated with glycolysis. However, in our network it is most closely associated with genes assigned to pyruvate metabolism and the glyoxylate cycle. The neighbors of *FBP1 *in this query include *ICL1*, *MLS1*, *SFC1*, *PCK1 *and *IDP3*. With the exception of *IDP3*, the promoters of all of these genes (including *FBP1*) have at least one upstream activation sequence that can be classified as a carbon source-response element (CSRE), and that responds to the transcriptional activator Cat8p [14]. This set of genes is expressed under non-fermentative growth conditions in the absence of glucose, conditions characteristic of the diauxic shift [15]. Considering other genes in the vicinity of *FBP1 *in the combined pathway query we find that *ACS1*, *IDP2*, *SIP4*, *MDH2*, *ACH1 *and *YJL045w *have all been shown to have either CSRE-like activation sequences and/or to be at least partially Cat8p dependent [14]. The association among these Cat8p-activated genes persists when we estimate the FOCI network without including the data of DeRisi *et al*. [15], suggesting that this set of interactions is not merely a consequence of the inclusion of data collected from cultures undergoing diauxic shift.

The inclusion of a number of other genes in the carbohydrate metabolism subnetwork is consistent with independent evidence from the literature. For example, McCammon *et al*. [16] identified *YER053c *as among the set of genes whose expression levels changed in TCA cycle mutants.

Although many of the associations among groups of genes revealed in these subgraphs can be interpreted either in terms of the query pathways used to construct them or with respect to related pathways, a number of association have no obvious biological interpretation. For example, the tail on the left of the graph in Figure 3, composed of *LSC1*, *PTR2*, *PAD1*, *OPT2*, *ARO10 *and *PSP1 *has no clear known relationship.

### Locally distinct subgraphs

The analysis of metabolic pathways described above provides a test of the extent to which known pathways are represented in the FOCI graph. That is, we assumed some prior knowledge about network structure of subsets of genes and asked whether our estimated network is coherent *vis-à-vis *this prior knowledge. Conversely, one might want to find interesting and distinct subgraphs within the FOCI network without the injection of any prior knowledge and ask whether such subgraphs correspond to particular biological processes or functions. To address this second issue we developed an algorithm to compute 'locally distinct subgraphs' of the yeast FOCI coexpression network as detailed in the Materials and methods section. Briefly, this is an unsupervised graph-search algorithm that defines 'interestingness' in terms of local edge topology and the distribution of local edge weights on the graph. The goal of this algorithm is to find connected subgraphs whose edge-weight distribution is distinct from that of the edges that surround the subgraph; thus, these locally distinct subgraphs can be thought of as those vertices and associated edges that 'stand out' from the background of the larger graph as a whole.

We constrained the size of the subgraphs to be between seven and 150 genes, and used squared marginal correlation coefficients as the weighting function on the edges of the FOCI graph. We found 32 locally distinct subgraphs, containing a total of 830 genes (Table 2). Twenty-four out of the 32 subgraphs have consistent Gene Ontology (GO) annotation terms [17] with *p*-values less than 10^-5 ^(see Materials and methods). This indicates that most locally distinct subgraphs are highly enriched with respect to genes involved in particular biological processes or functions. Members of the 21 largest locally distinct subgraphs are highlighted in Figure 1. The complete list of subgraphs and the genes assigned to them is given in Additional data file 2.

The five largest locally distinct subgraphs have the following primary GO annotations: protein biosynthesis (subgraphs A and B); ribosome biogenesis and assembly (subgraph C); response to stress and carbohydrate metabolism (subgraph K); and sporulation (subgraph N). Several of these subgraphs show very high specificity for genes with particular GO annotations. For example, in subgraphs A and B approximately 97% (32 out of 33) and 95.5% (64 out of 67) of the genes are assigned the GO term 'protein biosynthesis'.

Subgraph P is also relatively large and contains many genes with roles in DNA replication and repair. Similarly, 21 of the 34 annotated genes in Subgraph F have a role in protein catabolism. Three medium-sized subgraphs (S, T, U) are strongly associated with the mitotic cell cycle and cytokinesis. Other examples of subgraphs with very clear biological roles are subgraph R (histones) and subgraph Z (genes involved in conjugation and sexual reproduction). Subgraph X contains genes with roles in methionine metabolism or transport.

Some locally distinct subgraphs can be further decomposed. For example, subgraph K contains at least two subgroups. One of these is composed primarily of genes encoding chaperone proteins: *STI1*, *SIS1*, *HSC82*, *HSP82*, *AHA1*, *SSA1*, *SSA2*, *SSA4*, *KAR2*, *YPR158w*, *YLR247c*. The other group contains genes primarily involved in carbohydrate metabolism. These two subgroups are connected to each other exclusively through *HSP42 *and *HSP104*.

Three of the locally distinct subgraphs - Q, W and CC - are composed primarily of genes for which there are no GO biological process annotations. Interestingly, the majority of genes assigned to these three groups are found in subtelomeric regions. These three subgraphs are not themselves directly connected in the FOCI graph, so their regulation is not likely to be simply an instance of a regulation of subtelomeric silencing [18]. Subgraph Q includes 26 genes, five of which (*YRF1-2*, *YRF1-3*, *YRF1-4*, *YRF1-5*, *YRF1-6*) correspond to ORFs encoding copies of Y'-helicase protein 1 [19]. Eight additional genes (*YBL113c*, *YEL077c*, *YHL050c*, *YIL177c*, *YJL225c*, *YLL066c*, *YLL067c*, *YPR204w*) assigned to this subgraph also encode helicases. This helicase subgraph is closely associated with subgraph P, which contains numerous genes involved in DNA replication and repair (see Figure 1). Subgraph W contains 10 genes, only one of which is assigned a GO process, function or component term. However, nine of the 10 genes in the subgraph (*PAU1*, *PAU2*, *PAU4*, *PAU5*, *PAU6*, *YGR294w*, *YLR046c*, *YIR041w*, *YLL064c*) are members of the seripauperin gene family [20], which are primarily found subtelomerically and which encode cell-wall mannoproteins and may play a role in maintaining cell-wall integrity [18]. Another example of a subgraph corresponding to a multigene family is subgraph CC, which includes nine subtelomeric ORFs, six of which encode proteins of the COS family. Cos proteins are associated with the nuclear membrane and/or the endoplasmic reticulum and have been implicated in the unfolded protein response [21].

As a final example, we consider subgraph FF, which is composed of seven ORFs (*YAR010c*, *YBL005w-A*, *YJR026w*, *YJR028w*, *YML040w*, *YMR046c*, *YMR051c*) all of which are parts of Ty elements, encoding structural components of the retrotransposon machinery [22,23]. This set of genes nicely illustrates the fact that delineating locally distinct groups can lead to the discovery of many interesting interactions. There are only six edges among these seven genes in the estimated FOCI graph, and the marginal correlations among the correlation measures of these genes are relatively weak (mean *r *~ 0.62). Despite this, the local distribution of edge weights in FOCI graph is such that this group is highlighted as a subgraph of interest. Locally strong subgraphs such as these can also be used as the starting point for further graph search procedures. For example, querying the FOCI network for immediate neighbors of the genes in subgraph FF yields three additional ORFs - *YBL101w-A*, *YBR012w-B*, and *RAD10*. Both *YBL101w-A *and *YBR012w-B *are Ty elements, whereas *RAD10 *encodes an exonuclease with a role in recombination.

## Discussion

### Comparisons with other methods

Comparing the performance of different methods for analyzing gene-expression data is a difficult task because there is currently no 'gold standard' to which an investigator can turn to judge the correctness of a particular result. This is further complicated by the fact that different methods employ distinct representations such as trees, graphs or partitions that cannot be simply compared. With these difficulties in mind, we contrast and compare our FOCI method to three popular approaches for gene expression analysis - hierarchical clustering [1], Bayesian network analysis [10] and relevance networks [7,24,25]. Like the FOCI networks described in this report, both Bayesian networks and relevance networks represent interactions in the form of network models, and can, in principle, capture complex patterns of interaction among variables in the analysis. Relevance networks also share the advantage with FOCI networks that, depending on the scoring function used, they can be estimated efficiently for very large datasets.

### Comparison with relevance networks

Relevance networks are graphs defined by considering one or more scoring functions and a threshold level for every pair of variables of interest. Pairwise scores that rise above the threshold value are considered significant and are represented by edges in the graph; interactions below this threshold are discarded [25]. As applied to gene-expression microarray data, the scoring functions used most typically have been mutual information [7] or a measure based on a modified squared sample correlation coefficient [24]).

We estimated a relevance network for the same 5007-gene dataset used to construct the FOCI network. The scoring function employed was  with a threshold value of ± 0.5. The resulting relevance network has 13,049 edges and a GCC with 1,543 vertices and 12,907 edges. The next largest connected subgraph of the relevance network has seven vertices and seven edges. There are a very large number of connected subgraphs (3,341) that are composed of pairs or singletons of genes.

To compare the performance of the relevance network with the FOCI network we used the pathway query approach described above to test the coherence of the 38 metabolic pathways described previously. Of the 38 metabolic pathways tested, nine have significant coherence values in the relevance network. These coherent pathways include: glycolysis/gluconeogenesis, the TCA cycle, oxidative phosphorylation, ATP synthesis, purine metabolism, pyrimidine metabolism, methionine metabolism, amino sugar metabolism, starch and sucrose metabolism. Two of these pathways - amino sugar metabolism and starch and sucrose metabolism - are not significantly coherent in the FOCI network. However, there are 12 metabolic pathways that are coherent in the FOCI network but not coherent in the relevance network. On balance, the FOCI network model provides a better estimator of known metabolic pathways than does the relevance network approach.

### Comparison with hierarchical clustering and Bayesian networks

To provide a common basis for comparison with hierarchical clustering and Bayesian networks, we explored the dataset of Spellman *et al*. [26] which includes 800 yeast genes measured under six distinct experimental conditions (a total of 77 microarrays; this data is a subset of the larger analysis described in this paper). Spellman *et al. *[26] analyzed this dataset using hierarchical clustering. Friedman *et al. *[10] used their 'sparse candidate' algorithm to estimate a Bayesian network for the same data, treating the expression measurements as discrete values. For comparison with Bayesian network analysis we referenced the interactions highlighted in the paper by Friedman *et al*. and the website that accompanies their report [27]. For the purposes of the FOCI analysis we reduced the 800 gene dataset to 741 genes for which there were no more than 10 missing values. We conducted a FOCI analysis on these data using a partial correlation threshold of 0.33. The resulting FOCI network had 1599 edges and a GCC of 700 genes (the 41 other genes are represented by subgraphs of gene pairs or singletons).

On the basis of hierarchical clustering analysis of the 800 cell-cycle-regulated genes, Spellman *et al. *[26] highlighted eight distinct coexpressed clusters of genes. They showed that most genes in the clusters they identified share common promoter elements, bolstering the case that these clusters indeed correspond to co-regulated sets of genes (see [26] for description and discussion of these clusters).

Applying our algorithm for finding locally distinct subgraphs to the FOCI graph based on these same data (with size constraints min = 7, max = 75) we found 10 locally distinct subgraphs. Seven of these subgraphs correspond to major clusters in the hierarchical cluster analysis (the MCM cluster of Spellman *et al. *[26] is not a locally distinct subgraph). At this global level both FOCI analysis and hierarchical clustering give similar results. While the coarse global structure of the FOCI and hierarchical clustering are similar, at the intermediate and local levels the FOCI analysis reveals additional biologically meaningful interactions that are not represented in the clustering analysis. An example of interactions at an intermediate scale involves the clusters referred to as Y' and CLN2 in Spellman *et al. *[26] Genes of the CLN2 cluster are involved primarily in DNA replication. The Y' cluster contains genes known to have DNA helicase activity. The topology of the FOCI network indicates that these are relatively distinct subgraphs, but also highlights a number of weak-to-moderate statistical interactions between the Y' and CLN2 genes (and almost no interactions between the Y' genes and any other cluster). Thus the FOCI network estimate provides inference of more subtle functional relationships that cannot be obtained from the clustering family of methods.

An example at a more local scale involves the MAT cluster of Spellman *et al. *[26] This cluster includes a core set of genes whose products are known to be involved in conjugation and sexual reproduction. In the FOCI network one of the locally distinct subgraphs is almost identical to the MAT cluster, and includes *KAR4*, *STE3*, *LIF1*, *FUS1*, *SST2*, *AGA1*, *SAG1*, *MFα2 *and *YKL177W *(*MFα1 *is not included in the FOCI analysis because there were more than 10 missing values). The FOCI analysis additionally shows that this set of genes is linked to another subgraphs that includes *AGA2*, *STE2*, *MFA1*, *MFA2 *and *GFA3*. This second set of genes are also involved in conjugation, sexual reproduction, and pheromone response. *AGA1 *and *AGA2 *form the bridge between these two subgraphs (the proteins encoded by these two genes, Aga1p and Aga2p, are subunits of the cell wall glycoprotein *α*-agglutinin [28]). These two sets of genes therefore form a continuous subnetwork in the FOCI analysis, whereas the same genes are dispersed among at least three subclusters in the hierarchical clustering. We interpret the difference as resulting from the fact that the FOCI network can include relatively weak interactions among variables, as long as the variables are not first order conditionally independent. For example, the marginal correlation between *AGA1 *and *AGA2 *is only 0.63, between *AGA1 *and *GFA1 *is 0.59, and between *AGA2 *and *MFA1 *only 0.61. Hierarchical clustering or other analyses based solely on marginal correlations will typically fail to highlight such relatively weak interactions among genes.

Because hierarchical clustering constrains relationships to take the form of strict partitions or nested partitions, this type of analysis seems best suited to highlight the overall coarse structure of co-regulatory relationships. The FOCI method, because it admits a more complex set of topological relationships, is well suited to capturing both global and local structure of transcriptional interactions.

Graphical models, like the FOCI method, exploit conditional independence relationships to derive a model that can be represented using a graph or network structure. Unlike the FOCI model, general graphical models represent a complete factorization of a multivariate distribution. In the case of Bayesian networks it is also possible to assign directionality to the edges of the network model. However, these advantages come at the cost of complexity - Bayesian networks are costly to compute - and generally this complexity scales exponentially with the number of vertices (genes). The estimation of a FOCI network is computationally much less complex than the estimation of a Bayesian network. Both methods allow for a richer set of potential interactions among genes than does hierarchical clustering. We therefore expect that both methods should be able to highlight biologically interesting interactions, at both local and global scales. Friedman *et al. *[10] analyzed the 800-gene dataset of Spellman *et al*. [26] and highlighted a number of relationships that are assigned high confidence in their analysis. Relationships that were recovered under both a multinomial and Gaussian model include *STE2*-*MFA2*, *CTS1*-*DSE2*(*YHR143w*), *OLE1*-*FAA4*, *KIP3*-*MSB1*, *SHM2*-*GCV2*, *DIP5*-*ARO9 *and *SRO4*-*YOL007c*. All of these relationships, with the exception of *SRO5*-*YOL007c*, are present in the FOCI analysis of the same data.

Comparisons of the local topology of each network, based on examining the edge relationships for a number of query genes, suggests that the FOCI and Bayesian networks are broadly similar. There are of course, examples of biologically interpretable interactions that are present in the FOCI analysis but not in the Bayesian network and vice versa. For example, using a multinomial model, Friedman *et al. *demonstrated an interaction between *ASH1 *and *FAR1*, both of which are known to participate in the mating type switch in yeast. This relationship is absent in the FOCI network. Similarly, the relationship between *AGA1 *and *AGA2 *that is highlighted in the FOCI analysis does not appear in the multinomial Bayesian network analysis.

### Review of FOCI assumptions

As with all analytical tools, careful consideration of the assumptions underlying the FOCI network method is necessary to understand the limits of the inferences one can draw. For example, our current framework limits consideration to linear relationships as measured by correlations and partial correlations. These assumptions may be relaxed, allowing for other types of distributions and relationships among variables (for example, monotone and curvilinear relationships), but there is an inevitable trade-off to be made in terms of computational complexity and statistical power. However, as seen in our analysis, many biologically interesting relationships among gene expression measures appear to be approximately linear. Biologically speaking, it is important to keep in mind that the graphs resulting from a FOCI analysis of gene-expression measurements should properly be considered coexpression or co-regulation networks and not genetic regulatory networks *per se*. While the clusters and patterns of coexpression summarized by the FOCI network may result from particular regulatory dynamics, no causal hypothesis of regulatory interaction is implied by the network.

## Conclusions

Biology demands that the analytical tools we use for functional genomics should be able to capture and represent complex interactions; practical considerations stemming from the magnitude and scope of genomic data require the use of techniques that are computable and relatively efficient. The FOCI framework we have used for representing genomic coexpression patterns in terms of a weighted graph satisfies both these constraints. FOCI networks are readily computable, even for very large datasets. Comparisons with known metabolic pathways show that many key biological interactions are captured by FOCI networks, and the algorithm we provide for finding locally distinct subgraphs provides a mechanism for discovering novel associations based on local graph topology. The subgraphs and patterns of interactions that we are able to demonstrate based on such analyses are strongly consistent with known biological processes and functions, indicating that the FOCI network method is a powerful tool for summarizing biologically meaningful coexpression patterns. Furthermore, the kinds of interactions captured by network analysis are typically more natural than the clustering family of analyses where biased and unstable results can be forced by the algorithm. Secondary analysis based on the network properties also reveal additional subtle structure. For example, our procedure for finding locally distinct subgraphs reveals associated genes whose pairwise interactions may be globally weak but relatively strong compared to their local interactions. While the results reported here focus on the analysis of gene expression measurements, the FOCI approach can be applied to any type of quantitative data making it a generally suitable technique for exploratory analyses of functional genomic data.

## Materials and methods

### A statistical/geometrical model for estimating coexpression networks

The approach we employ to estimate coexpression networks is based on a general statistical technique we have developed for representing the associations among a large number of variables in terms of a weighted, undirected graph. The technique is based on the consideration of so-called 'first-order' conditional independence relationships among variables, hence we call the graphs that result from such analyses first-order conditional independence, FOCI, networks. The network representation that results from a FOCI analysis also has a dual geometrical interpretation in terms of proximity relationships defined with respect to the geometry of correlations and partial correlations. We outline the statistical and geometrical motivations underlying our approach below.

### First-order conditional independence networks

A FOCI network is a graph, *G *= {*V*,*E*}, where the vertex set, *V*, represents the variables of interest and the edge set, *E*, represents interactions among the variables. *e*_*ij *_is an edge in *G*, if and only if there is no other variable in the analysis, *k*(*k *≠ *k *≠ *k*) such that  or , where



 is a modified partial correlation between *i *and *j *conditioned on *k*.  takes values in the range -1 ≤  ≤ 1.  is approximately zero when *i *and *j *are independent conditional on *k*.  is positive when the marginal correlation, *ρ*_*ij*_, and the standard partial correlation, *ρ*_*ij*.*k*_, agree in sign, and is negative otherwise. Cases where the marginal and conditional correlations are of opposite sign are examples of 'Simpson's paradox', which usually indicates that there is a lurking or confounding effect of the conditioning variable (see [29] for a general discussion of such relationships).

While true biological interactions may sometimes lead to inverted conditional associations, their interpretation can be complicated; therefore in the analysis presented above, we did not connect edges when the relationships became inverted. However, one can also keep such edges for subsequent analysis if there is reasonable functional justification. When such sign-reversed edges are ignored, we will call this the sign-restricted FOCI network. This definition means that variables *i *and *j *are connected in the FOCI network if there is no other variable in the analysis for which *i *and *j *are conditionally independent or which causes an association reversal. Because we restrict the conditioning set to single variables, these are so called 'first-order' conditional interactions (marginal correlations correspond to zero-order conditional interactions; partial correlations given two conditioning variables are second-order conditional interactions, etc). If *i *and *j *are conditionally independent given *k *we write this as (*i *⊥ *j*|*k*). Using an information theoretic interpretation suggested by Lauritzen [9], the statement (*i *⊥ *j*|*k*) implies that if we observe the variable *k*, there is no additional information about *i *that we gain by also observing *j *(and vice versa). Because the edges of the FOCI network indicate pairs of variables that are not conditionally independent, one can interpret the FOCI graph as a summary of all the pairwise interactions that can not be 'explained away' by any other single variable in the analysis.

Unlike standard graphical models, a FOCI network does not represent a factorization of a multivariate distribution into the product of simpler distributions. However, below we show that a sign-restricted FOCI graph has a unique geometric interpretation in terms of proximity relationships in the multidimensional space that represents the correlations among variables. This geometric interpretation suggests that the FOCI model should be a generally useful approach for exploratory analyses of very high-dimensional datasets.

Our FOCI approach is similar to a framework developed by de Campos and Huete [30] for estimating belief networks. These authors developed an algorithm based on the application of zero- and first-order conditional independence test to learn the 'prior skeleton' of a Bayesian network, followed by a refinement procedure that uses higher-order interactions sparingly.

### Geometrical model of first-order conditional independence

Above we described the FOCI network model in statistical terms. Here we provide a geometrical interpretation of FOCI graphs. We show that a FOCI network is equivalent to a proximity graph of the variables of interest (genes in the current analysis). More specifically, we demonstrate that a sign-restricted FOCI network is a 'Gabriel graph' in the geometric space that represents the relationships among the variables.

A Gabriel graph, introduced by Gabriel and Sokal [31], is a type of proximity graph. Let *B*(*x*,*r*) denote an open *n*-sphere centered at the point *x *with radius *r*, and let *d*(*p*,*q*) denote the Euclidean distance function. Given a set of points, *P *= {*p*_1 _*p*_2_, ..., *p*_*n*_}, in an *n*-dimensional Euclidean space, (*p*_*i*_, *p*_*j*_) is an edge in the Gabriel graph if no other point, *p*_*k *_(*i *≠ *k*, *j *≠ *k*) in *P *falls within the diameter sphere defined by *B*((*p*_*i *_= *p*_*j*_)/2, *d*(*p*_*i*_, *p*_*j*_)/2). That is, *p*_*i *_and *p*_*j *_are connected in the Gabriel graph if no other point falls within the sphere that has the chord *p*_*i*_, *p*_*j *_as its diameter [32].

### Geometry of marginal and partial correlations and conditional independence

One can represent random variables as vectors in the space of the observations (often called object space or subject space [33,34]). In such a representation, a set of mean centered and standardized variables correspond to unit vectors whose heads lie on the surface of an *n*-dimensional hypersphere (where *n *is the number of observations). In this representation, the correlation between two random variables, *x *and *y*, is given by the cosine of the angle between their vectors. We will refer to this construction as the 'correlational hypersphere'. The partial correlation between *x *and *y *given *z *is equivalent to the cosine of the angle between the residual vectors obtained by projecting *x *and *y *onto *z*. The vectors *x*, *y *and *z *form the vertices, A, B, and C, of a spherical triangle on that hypersphere with associated angles *γ*, *λ*, and *φ*. Then, *ρ*_*xy*.*z *_= cos(*φ*), *ρ*_*xz*.*y *_= cos(*λ*), and *ρ*_*yz*.*x *_= cos(*γ*) [35]. Given this geometric construction of partial correlations in terms of spherical triangles, conditional independence, defined as *ρ*_*xy*.*z *_= 0 for the multivariate normal, is obtained when cos(*φ*) = 0 (that is, when the *φ *= *π*/2). The set of *z *vectors that satisfy this condition defines a circle (actually a hypersphere of dimension *n *- 1) on the hypersphere whose diameter is the spherical chord between *x *and *y*. If the projection of *z *onto the hypersphere lies outside of this circle then *ρ*_*xy*.*z *_is positive, inside the circle *ρ*_*xy*.*z *_is negative (with *ρ*_*xy*.*z *_= -1 along the chord between *x *and *y*).

The sign-restricted FOCI network construction corresponds to the graph obtained by connecting variables *i *and *j *only if no third variable falls within the diameter sphere defined by *i *and *j *on the correlational hypersphere, or by the diameter sphere defined by *i *and -*j *when *r*_*ij *_< 0 (allowing for deviations due to sampling). This is the same criteria of proximity that defines a Gabriel graph. A FOCI graph is therefore a summary of relative proximity relationships among the variables of interest, defined with respect to the geometry of correlations when restricted to the cases when the partial correlation signs are consistent with the marginal correlations.

### FOCI network algorithm

A simple algorithm for estimating a network based on first-order conditional independence relationships is described below. The results of this algorithm can be represented as a graph where the vertices represent the variables of interest (genes) and the edges represent interactions among variables that show at least first-order conditional dependence. A library of functions for estimating FOCI networks, implemented in the Python programming language, is available from the authors on request.

We use vanishing partial correlations [8,36] to test whether pairs of genes are conditionally independent given any other single variable in the analysis. Strictly speaking, if the data are not multivariate normal, then zero partial correlations need not imply conditional independence, but rather conditional uncorrelatedness [37]. However, regardless of distributional assumptions, zero partial correlations among variates are of interest as long as the relationship between the variables has a strong linear component [38].

#### FOCI algorithm

1. **Estimate marginal associations. **For a set of *p *variables, indexed by *i *and *j*, calculate the *p *× *p *correlation matrix, *C*, where *C*_*i*,*j *_= corr(*i, j*) for all *i*, *j*; *i *= 1...*p*, *j *= 1...*p*.

2. **Construct saturated graph. **Construct a *p *× *p *adjacency matrix, *G*. Let *G*_*i*,*j *_= 1 for all *i*, *j*.

3. **Prune zero-order independent edges. **For each pair of variables, (*i*, *j*), if *C*_*i*,*j *_<*T*_*crit *_(or some appropriately chosen function, *f*(*C*_*i*,*j*_) <*T*_*crit*_), where *T*_*crit *_is a threshold value for determining marginal/conditional independence (see below), then set *G*_*i*,*j *_= 0. *G *defines a marginal independence graph.

4. **Estimate first-order relationships. **For each pair of variables (*i*, *j*) in *G *calculate , the minimum partial correlation between *i *and *j*, conditioned on each of the other variables in the analysis taken one at a time.  for all *k *such that *i *≠ *k *and *j *≠ *k *and (*i*, *k*) and (*j*, *k*) are both edges in *G*.  is the sample modified partial correlation coefficient as defined in equation (1).

5. **Prune first-order independent edges. **If  <*T*_*crit *_(or *f*() <*T*_*crit *_then set *G*_*i*,*j *_= 0.

The resulting adjacency matrix *G*, can be represented as an undirected graph, with *p *vertices, whose edge set is defined by the non-zero elements in *G*. The edges of this graph can be represented as either unweighted (all edges having equal weight) or with weights defined by some function of corr(*i, j*) or . If we assume multivariate normality we can use Fisher's z-transformation [39] to normalize the expected distribution of correlation/partial correlations and use standard tables of the normal distribution to define *T*_*crit *_for a given edge-wise false-positive rate. Alternatively, one can define *T*_*crit *_by other methods such as via permutation analysis to define a null distribution for . While the FOCI approach requires that one define a critical threshold for determining conditional independence, this threshold is in theory a function of the sample size and the null distribution of  rather than the somewhat fuzzier distinction between 'strong' and 'weak' correlation that most pairwise network estimation approaches require.

### Estimating the yeast FOCI coexpression network

We used the FOCI network algorithm to estimate a coexpression network for the budding yeast, *Saccharomyces cerevisae*. The data used in our analysis are drawn from publicly available microarray measurements of gene expression described in DeRisi *et al. *[15], Chu *et al. *[40] and Spellman *et al. *[26]. These data represent relative measurements of gene expression taken at different points in the cell cycle in yeast cultures synchronized using a variety of different mechanisms [26] or in the context of specific physiological process such as diauxic shift [15] or sporulation [40]. The data were log_2_-transformed, duplicate and missing data were removed and any ORFs listed as 'dubious' in the *Saccharomyces *Genome Database as of December 2003 were filtered out. The final dataset consisted of expression measurements for 5,007 ORFs represented by 87 microarrays (see Rifkin *et al. *[41] for a full description of the pretreatment of these data). The mean centered data were treated as continuous variables for the purposes of our analysis.

Microarray measurements, especially spotted microarrays, are subject to a variety of systematic effects such as those due to dye biases and print-tip effects, and a number of methods have been devised to normalize and correct for such biases [42,43]. However, the data analyzed here include both spotted DNA microarray measurements and expression measurements based on Affymetrix arrays (experiments of Cho *et al. *[44] as reported by Spellman *et al. *[26]), making it difficult to apply a consistent correction. Another consideration is that the assemblage of experiments considered by Spellman *et al. *[26], have been frequently used to illustrate the utility of new analytical methods [7,10,45]. To facilitate comparison with previous reports we have chosen to analyze these data without any transformations other than the log-transformation and mean-centering described above.

As noted above, zero partial correlations are exactly equivalent to conditional independence only for multivariate normal distributions. However, from the perspective of exploratory analyses, the more important assumption is that the relationships among the gene expression measures are predominantly linear. We tested each of these assumptions as follows. We used a Cramer-von Mises statistic [46] to test for the normality of each vector of gene expression measurements. Approximately 59% of the univariate distributions of the variables are consistent with normality (*p *< 0.05). While a majority of the univariate distributions are approximately normal, a significant proportion of the trivariate distributions are clearly not multivariate normal. As a crude test of linearity for bivariate relationships we calculated linear regressions for 10,000 random pairs of gene expression measures (randomly choosing one of the pair as the dependent variables), and performed runs tests [47] for randomness of the signs of the residuals from each regression. Significant deviations from non-linearity in the bivariate relationships should manifest themselves as non-random runs of positive or negative residuals. For approximately 95% of the runs tests we can not reject the null hypothesis of randomness in the signs of the residuals (*p *< 0.05). We therefore conclude that the assumption of quasi-linearity is valid for a large number of the pairwise relationships.

Given these observations, in order to define an appropriate partial correlation threshold, *T*_*crit*_, for these data we considered both permutation tests and false-positive rates based on asymptotic expectations for the distribution of first-order partial correlations (see above). Permutation tests were carried out by independently randomizing the values for each gene expression variable such that each gene had the same mean and variance as its original observation vector, but both the marginal and partial correlations had an expected value of zero. We then sampled 1,000 such randomized variables and examined the distribution of  for every pair of variables in this sample. For *p *≤ 0.001 the permutation test indicates a value of *T*_*crit *_~ 0.3. The asymptotic threshold for *p *≤ 0.001 based on Fisher's z-transform is *T*_*crit *_~ 0.3. We used the slightly more conservative value of *T*_*crit *_~ 0.34.

### Metabolic pathways

We used 38 metabolic pathways as documented in KEGG release 29.0, January 2004 [48,49] to test the biological relevance of the estimated yeast coexpression network. These pathways are listed in Table 1. In our analysis we only considered metabolic pathways for which more than 10 pathway genes were represented in the gene expression dataset described above. The metabolic pathways we studied are not independent, as there are a number of genes whose products participate in two or more metabolic processes. However, for the purposes of the present analysis we have treated each pathway as independent.

### Testing the coherence of pathways using pathway queries

We used the following method to compare our FOCI network to the metabolic pathways from KEGG. We say that a subset of vertices, *H*, is two-step connected in the graph *G *if no vertex in *H *is more than two edges away from at least one other element of *H*. Given a set of genes assigned to a pathway (the query genes), we computed the set of two-step connected subgraphs for the query genes in the GCC of our yeast coexpression network. This procedure yields one or more subgraphs that are composed of query (pathway) genes plus non-query genes that are connected to at least two pathway genes. We used two steps as a criterion for our pathway queries because our estimate of the distribution of path distances (Figure 2b) indicated that more than 99% of gene pairs in our network are separated by a distance greater than two steps. Therefore, two-step connected subgraphs in our coexpression network represent sets of genes which are relatively close to each other with respect to the topology of the graph as a whole.

Suppose we have a set of query genes from a known pathway denoted as *P *= {g_1_,*g*_2_,...*g*_*k*_}. We construct the two-step connected graph of the elements of *P *from our FOCI estimated network denoted as *F*_*P *_⊃ *P*. That is, *F*_*P *_is a subgraph from the FOCI network that contains elements of *P *and its neighbors according to the two-step connected criteria described above. *F*_*P *_may itself be composed of one or more connected components. We define *F*_*Pmax *_as the connected component of *F*_*P *_that has the greatest overlap with *P*. If the FOCI network was completely coherent with respect to *P*, than *F*_*P *_should constitute a single connected component (that is, *F*_*Pmax *_= *F*_*P*_) whose vertex set completely overlaps *P *(that is, |*F*_*p *_∩ *P*| = |*P*|). For cases in which the query pathway is less than perfectly represented in the estimated network we measure the degree of coherence as |*F*_*Pmax *_∩ *P*| / |*P*|). We refer this ratio the 'coherence value' of the pathway *P *in the network of interest. However, we note that in a completely connected graph (that is, every vertex is connected to every other vertex), every possible pathway query would be maximally coherent but so would any random set of genes. It is therefore necessary to compare the coherence of a given pathway to the distribution of coherence values for random pathways composed of the same number of genes drawn from the same network. We estimated this distribution by using a randomization procedure in which we used 1,000 replicate random pathways to estimate the distribution of coherence values for pathways of different sizes. In Table 1, pathways that are significantly more coherent than at least 95% of random pathways are marked with an asterisk.

### Locally distinct subgraphs of coexpression networks

We describe an algorithm for extracting a set of 'locally distinct' subgraphs from an edge-weighted graph. We assume that the edge-weights of the graph are measures of the strength of association between the variables of the interest. We define a locally distinct subgraph as a subgraph in which all edges within the subgraph are stronger than edges that connect subgraph vertices to vertices not within the subgraph. Such subgraphs are 'locally distinct' because they are defined not by an absolute threshold on edge strengths, but rather by a consideration of the local topology of the graph and the distribution of edge weights. We describe an algorithm for finding locally distinct subgraphs below.

#### An algorithm for finding locally distinct subgraphs

Let *G *= {*V*, *E*} and *w*:*E *→ **R **be an edge-weighted graph where *w*(*e*) is the edge weight function, and |*V*| = *p *and |*E*| = *q*. Define an ordering on *E*, *O*(*E*) = (*e*_1_,*e*_2_,...,*e*_*q*_), such that *w*(*e*_*i*_) ≥ *w*(*e*_*j*_) for all *i *≤ *j *(that is, order the edges from strongest to weakest). Let *G*(*τ*)*= *{*V*, *E*(*τ*)} be a subgraph of *G *obtained by deleting all edges, *e*, such that *w*(*e*) <*e*_*τ*_. *G*(*τ*) an edge-level graph. Also let  denote the *k *connected components of *G*(*τ*). Let Ω = *C*_1 _∪ *C*_2 _∪ … *C*_*n*_. Define *L*_*α*,*ζ *_= {*l*_1_,*l*_2_,...,*l*_*m*_} where *l*_*i *_⊆ Ω, *l*_*i *_∩ *l*_*j *_=  (*i *≠ *j*) and *α *≤|*l*_*i*_|≤ *ζ*. That is, *L*_*α*,*ζ *_is a collection of disjoint subgraphs of *G*, where every *l*_*i *_is a connected component of some *G*(*τ*) and the size of *l*_*i *_is between *α *and *ζ*. We call the elements of *L*_*α*,*ζ *_the *α*,*ζ*-constrained locally distinct subgraphs of *G*. We say *L*_*α*,*ζ *_is optimal if |*l*_*i *_∪ *l*_*j *_… *l*_*m*_| is maximal and |*L*_*α*,*ζ*_| is minimal. Our goal is to find the optimal *L*_*α*,*ζ *_for the graph *G *given the constraints *α *and *ζ*. A simple algorithm for calculating the *L*_*α*,*ζ *_is as follows:

1. let *L *← , *i *= 0

2. while *i *≤ *q*:

3.  calculate *G*(*i*) and *C*_*i*_

4.  for  in *C*_*i*_:

5.   if  :

6.    for *l *in *L*:

7.     if  :

8.      *L *← *L *- {*l*}

9.     

10. *i *= *i *+ 1

11. *L*_*α*,*ζ *_← *L*

The algorithm is straightforward. At each iteration, *i*, we calculate the connected components of the edge-level graph, *G*(*i*), and add those components which satisfy the size constraints to the candidate list *L*. Lines 6-8 of the algorithm serve to eliminate from *L *any non-maximal components.

### Biological significance of locally distinct subgraphs

We applied the locally distinct subgraph algorithm to our yeast FOCI coexpression network. We used pairwise marginal correlations as the edge-weighting function, and set the size constraints as *α *= 7, *ζ *= 150. The subgraph search given these constraints yielded 32 locally distinct subgraphs (see Table 2 and Additional data file 2). For each locally distinct subgraph found we used the SGD Gene Ontology (GO) term finder of the *Saccharomyces *Genome Database [50,51] to search the set of genes in each subgraph for significant shared GO terms. We excluded from the term finder search any genes for which no biological process or molecular function term was assigned. Table 2 summarizes the primary GO terms assigned to each subgraph and the number of genes labeled with that GO term is shown in parentheses. The *p*-values in Table 2 indicate the frequency at which one would expect to find the same number of genes assigned to the given GO term in a random assemblage of the same size.

## Additional data files

Additional data are available with the online version of this article. Additional data file 1 provides supplementary figures illustrating the connectivity distribution (on a log-log scale) of the estimated yeast FOCI network and additional examples of coherent subgraphs of the FOCI network generated by querying with known metabolic pathways. Additional data file 2 contains a table detailing each of the 32 locally distinct subgraphs generated from the yeast FOCI network via the unsupervised graph search algorithm described in the text. A listing is provided for each locally distinct subgraphs describing yeast ORFs assigned to that subgraph and the Yeast GO Slim annotations associated with each ORF.

## Supplementary Material

Additional data file 1 Supplementary figures illustrating the connectivity distribution (on a log-log scale) of the estimated yeast FOCI network and additional examples of coherent subgraphs of the FOCI network generated by querying with known metabolic pathways Click here for additional data file

Additional data file 2 A table detailing each of the 32 locally distinct subgraphs generated from the yeast FOCI network via the unsupervised graph search algorithm described in the text Click here for additional data file

## Figures and Tables

**Table 1:** Summary of queries for 38 metabolic pathways against the yeast FOCI coexpression network

Pathway	Number of genes(in KEGG)	Size of largest coherent subnetwork(s)
**Carbohydrate metabolism**		
Glycolysis/gluconeogenesis	41 (47)	18*
Citrate cycle (TCA cycle)	27 (30)	18*
Pentose phosphate pathway	20 (27)	6*
Fructose and mannose metabolism	39 (46)	4
Galactose metabolism	25 (30)	8*
Ascorbate and aldarate metabolism	11 (13)	3
Pyruvate metabolism	32 (34)	8*
Glyoxylate and dicarboxylate metabolism	12 (14)	6*
Butanoate metabolism	27 (30)	7*
		
**Energy metabolism**		
Oxidative phosphorylation	53 (76)	31*
ATP synthesis	21 (30)	7*
Nitrogen metabolism	24 (27)	3
		
**Lipid metabolism**		
Fatty acid metabolism	13 (17)	3
		
**Nucleotide metabolism**		
Purine metabolism	87 (99)	34*
Pyrimidine metabolism	72 (80)	15*
Nucleotide sugars metabolism	11 (14)	2
		
**Amino acid metabolism**		
Glutamate metabolism	25 (27)	3
Alanine and aspartate metabolism	26 (27)	7*
Glycine, serine and threonine metabolism	36 (42)	7*
Methionine metabolism	13 (14)	6*
Valine, leucine and isoleucine biosynthesis	15 (16)	10*
Lysine biosynthesis	16 (20)	3
Lysine degradation	26 (30)	4
Arginine and proline metabolism	20 (24)	5*
Histidine metabolism	20 (25)	3
Tyrosine metabolism	27 (34)	2
Tryptophan metabolism	20 (25)	2
Phenylalanine, tyrosine and tryptophan biosynthesis	21 (23)	6*
		
**Metabolism of complex carbohydrates**		
Starch and sucrose metabolism	118 (139)	29
N-Glycans biosynthesis	43 (49)	13*
O-Glycans biosynthesis	18 (20)	2
Aminosugars metabolism	16 (20)	2
Keratan sulfate biosynthesis	18 (20)	2
		
**Metabolism of complex lipids**		
Glycerolipid metabolism	56 (68)	12*
Inositol phosphate metabolism	87 (103)	10
Sphingophospholipid biosynthesis	101 (118)	11
		
**Metabolism of cofactors and vitamins**		
Vitamin B6 metabolism	11 (14)	2
Folate biosynthesis	14 (17)	1

The values in the second column represent the number of pathway genes represented in the GCC of the yeast FOCI graph, with the total number of genes assigned to the given pathway in parentheses. The third column indicates the number of pathway genes in the largest coherent subgraph resulting from each pathway query. Pathways represented by coherent subgraphs that are significantly larger than are expected at random (*p *< 0.05) are marked with asterisks.

**Table 2:** Summary of locally distinct subgraphs of the yeast FOCI coexpression network

Subgraph	Number of genes	Number unkown	Major GO terms	*p*-value
A	33	0	Protein biosynthesis (32)	**1.82e-30**
B	67	2	Protein biosynthesis (64)	**2.20e-61**
C	124	26	Ribosome biogenesis and assembly (74)	**2.10e-89**
D	10	0	Glycolysis/gluconeogenesis (8)	**6.29e-20**
E	7	1	Carboxylic/organic acid metabolism (4)	**5.07e-05**
F	41	7	Ubiquitin dependent protein catabolism (21)	**1.37e-31**
G	14	4	Cell organization and biogenesis (7)	1.60e-04
H	7	0	Main pathways of carbohydrate metabolism (4)	**2.46e-07**
I	13	0	Electron transport (7)	**2.00e-15**
J	13	0	Glutamate biosynthesis/TCA cycle (4)	**7.09e-10**
K	71	25	Response to stress (17); carbohydrate metabolism (13)	**3.94e-11**
L	10	4	Response to stress (2)	3.35e-02
N	149	51	Sporulation (27)	**2.23e-29**
M	5	2	Mitochondrial matrix (5); mitochondrial ribosome (4)	**2.83e-09**
O	7	2	Meiosis (4)	**3.77e-07**
P	52	13	Cell proliferation (32); DNA replication and chromosome cycle (28)	**1.12e-28**
Q	26	21	Telomerase-independent telomere maintenance (5)	**1.82e-14**
R	7	0	Chromatin assembly/disassembly (7)	**4.25e-18**
S	14	5	Cell wall (4); bud (4)	**4.47e-05**
T	24	8	Cell proliferation (15); mitotic cell cycle (9)	**6.54e-16**
U	21	4	Cell separation during cytokinesis (4); cell proliferation (9); cell wall organization and biogenesis (5)	**5.27e-10**
V	12	4	Metabolism (7)	2.48e-02
W	10	9	Nine of ten are members of the seripauperin gene family	NA
X	9	0	Sulfur amino acid metabolism (6); amino acid metabolism (3)	**3.33e-13**
Y	7	1	Cell growth and maintenance (6)	7.50e-04
Z	19	2	Conjugation with cellular fusion (13)	**1.82e-21**
AA	8	4	Biotin biosynthesis (2)	**1.81e-06**
BB	7	0	Response to abiotic stimulus (2)	1.48e-02
CC	9	5	Six of nine members belong to COS family of subtelomerically encoded proteins	NA
DD	18	7	Cell growth and/or maintenance (8)	4.43e-03
EE	11	3	Vitamin B6 metabolism (2)	**2.58e-05**
FF	7	0	Ty element transposition (7)	**6.01e-14**

The columns of the table summarize the total size of the locally distinct subgraph, the number of genes in the subgraph that are unannotated (according to the GO Slim annotation from the Saccharomyces Genome Database of December 2003), the primary GO term(s) associated with the subgraph, and a p-value indicating the frequency at which one would expect to find the same number of genes assigned to the given GO term in a random assemblage of the same size.
