# Supplementary material for: Estimating genomic coexpression networks using first-order conditional independence
Source: Genome Biol. 2004 Nov 30;5(12):R100. doi: 10.1186/gb-2004-5-12-r100 (PMC545795; doi:10.1186/gb-2004-5-12-r100)
Supplement: Additional data file 1 — Supplementary figures illustrating the connectivity distribution (on a log-log scale) of the estimated yeast FOCI network and additional examples of coherent subgraphs of the FOCI network generated by querying with known metabolic pathways [file gb-2004-5-12-r100-s1.pdf]

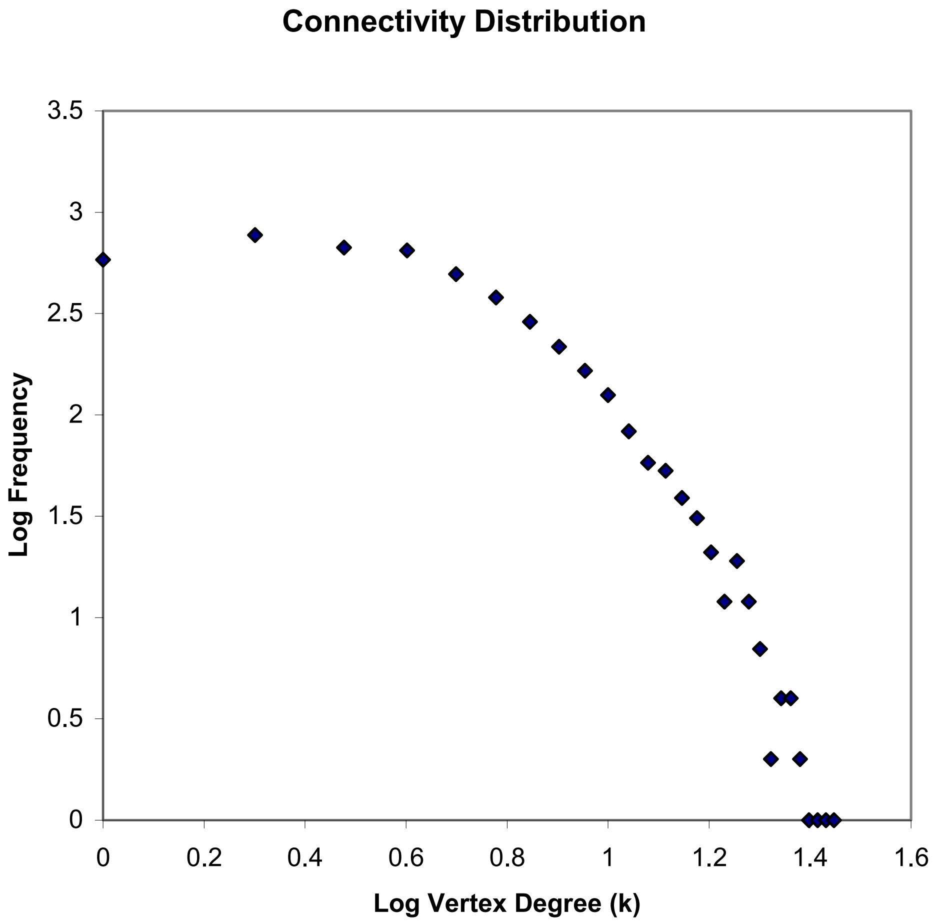


**Supplementary Figure 1.** Log-log plot of connectivity distribution for the estimated yeast FOCI network. This distribution does not conform to a simple power law distribution.

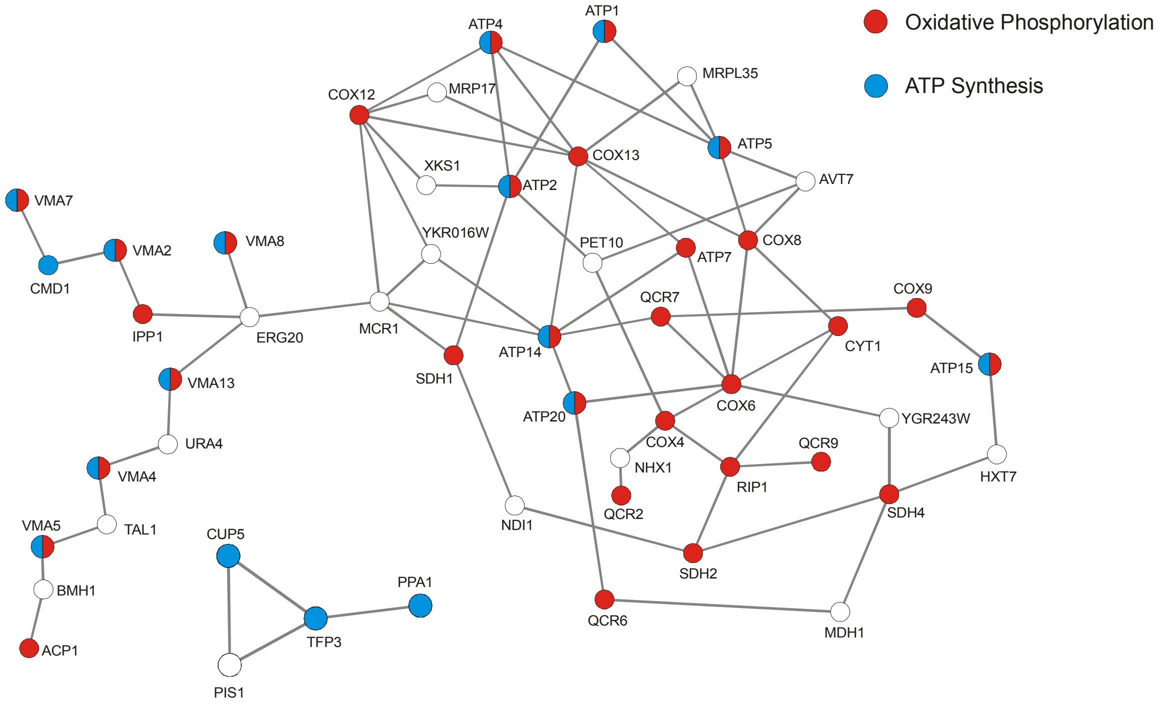


## Oxidative Phosphorylation and ATP Synthesis

**Supplementary Figure 2.** Coherent subnetworks generated by pathway queries on the oxidative phosphorylation and ATP synthesis pathways. There is a large amount of overlap in the genes assigned to each pathway, and this figure illustrates the single largest coherent subgraph for the Oxidative Phosphorylation pathway and an additional smaller subgraph composed primarily of genes assigned to the ATP synthesis pathway. Query genes are colored red (oxidative phosphorylation) and blue (ATP synthesis); vertices representing non-query genes are drawn in white.

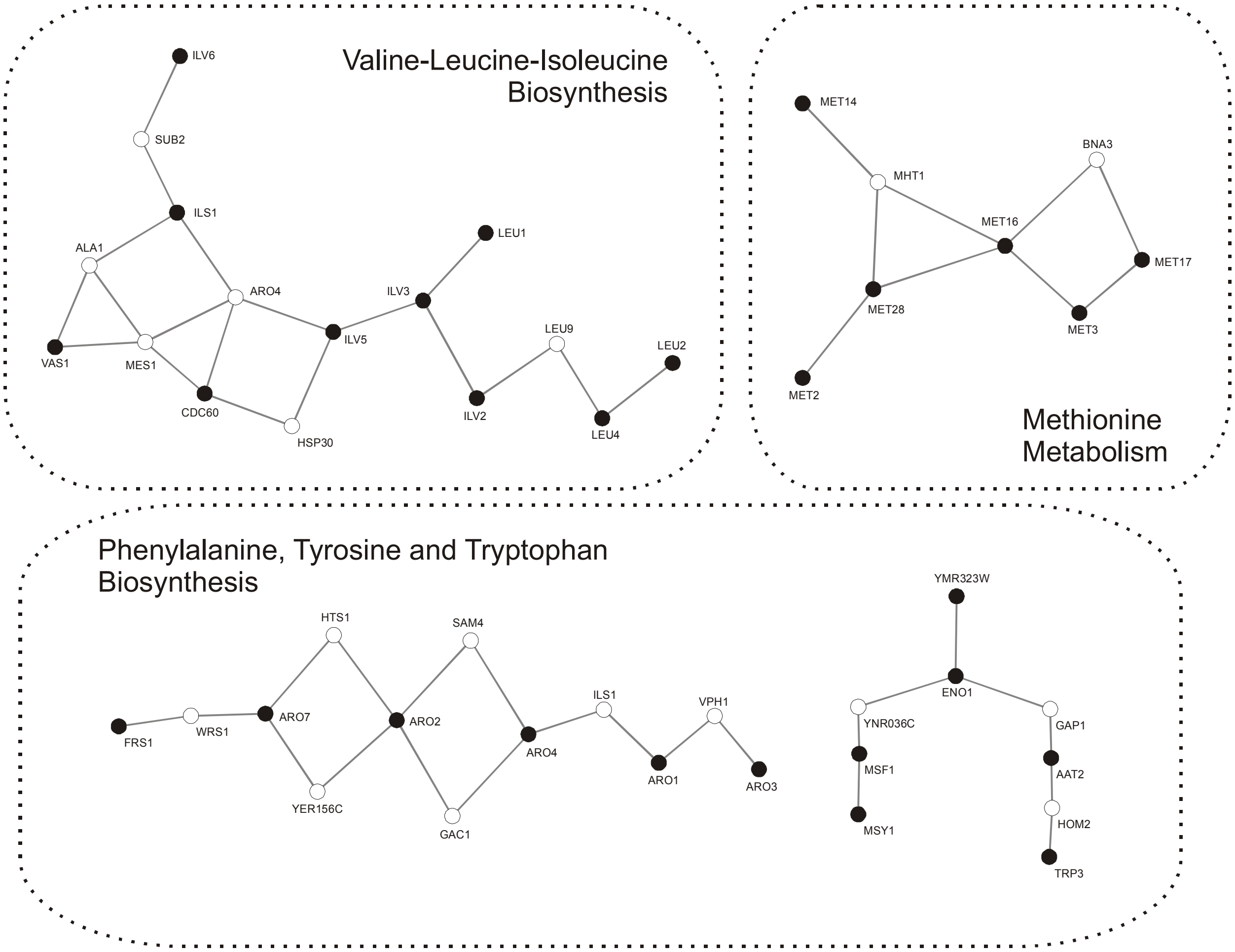


**Supplementary Figure 3.** Coherent subnetworks corresponding to pathway queries on three amino acid metabolism pathways: 1) valine-leucine-isoleucine biosynthesis; 2) methionine metabolism; and 3) phenylalanine, tyrosine and tryptophan biosynthesis. Filled nodes represent query genes; non-query nodes are shown in white. For the first two pathways we show only the single largest two-step connected subgraphs. The phenylalanine, tyrosine, and tryptophan query yielded two subnetworks each containing six query genes, both of which are illustrated.
