# Supplementary material for: Estimating genomic coexpression networks using first-order conditional independence
Source: Genome Biol. 2004 Nov 30;5(12):R100. doi: 10.1186/gb-2004-5-12-r100 (PMC545795; doi:10.1186/gb-2004-5-12-r100)
Supplement: Additional data file 2 — A table detailing each of the 32 locally distinct subgraphs generated from the yeast FOCI network via the unsupervised graph search algorithm described in the text [file gb-2004-5-12-r100-s2.pdf]

Locally Distinct Subgraphs
Each group listed below represents one of the locally distinct subgraphs extracted from the giant connected component of the yeast co-expression network using the graph search algorithm described in the text.

Note: the annotation used here corresponds to the Yeast GO Slim biological process annotation from SGD.

ORF	Gene Name	GO Slim
**Group A**		
YAL038W	CDC19	carbohydrate metabolism
YDR418W	RPL12B	protein biosynthesis
YLR029C	RPL15A	protein biosynthesis
YPL220W	RPL1A	protein biosynthesis
YBR191W	RPL21A	protein biosynthesis
YLR061W	RPL22A	protein biosynthesis
YGL031C	RPL24A	protein biosynthesis
YGR148C	RPL24B	protein biosynthesis
YOL127W	RPL25	protein biosynthesis
YLR344W	RPL26A	protein biosynthesis
YHR010W	RPL27A	protein biosynthesis
YGL103W	RPL28	protein biosynthesis
YBR031W	RPL4A	protein biosynthesis
YDR012W	RPL4B	protein biosynthesis
YLR340W	RPP0	protein biosynthesis
YDL081C	RPP1A	protein biosynthesis
YDL130W	RPP1B	protein biosynthesis
YDR382W	RPP2B	protein biosynthesis
YCR031C	RPS14A	protein biosynthesis
YOL040C	RPS15	protein biosynthesis
YDL083C	RPS16B	protein biosynthesis
YDR450W	RPS18A	protein biosynthesis
YLR441C	RPS1A	protein biosynthesis
YJL190C	RPS22A	protein biosynthesis
YGL189C	RPS26A	protein biosynthesis
YER131W	RPS26B	protein biosynthesis
YOR167C	RPS28A	protein biosynthesis
YDL061C	RPS29B	protein biosynthesis
YOR182C	RPS30B	protein biosynthesis
YLR167W	RPS31	protein biosynthesis
YJR145C	RPS4A	protein biosynthesis
YBR189W	RPS9B	protein biosynthesis
YBR118W	TEF2	protein biosynthesis

Known: 33, Unknown: 0, Not in annotation: 0

**Group B**		
YOL139C	CDC33	cell cycle
YLR150W	STM1	nuclear organization and biogenesis, response to stress
YPL037C	EGD1	protein biosynthesis

YLR075W	RPL10	protein biosynthesis
YPR102C	RPL11A	protein biosynthesis
YGR085C	RPL11B	protein biosynthesis
YEL054C	RPL12A	protein biosynthesis
YDL082W	RPL13A	protein biosynthesis
YKL006W	RPL14A	protein biosynthesis
YIL133C	RPL16A	protein biosynthesis
YNL069C	RPL16B	protein biosynthesis
YJL177W	RPL17B	protein biosynthesis
YOL120C	RPL18A	protein biosynthesis
YNL301C	RPL18B	protein biosynthesis
YBR084C-A	RPL19A	protein biosynthesis
YBL027W	RPL19B	protein biosynthesis
YGL135W	RPL1B	protein biosynthesis
YPL079W	RPL21B	protein biosynthesis
YER117W	RPL23B	protein biosynthesis
YGR034W	RPL26B	protein biosynthesis
YDR471W	RPL27B	protein biosynthesis
YIL018W	RPL2B	protein biosynthesis
YOR063W	RPL3	protein biosynthesis
YGL030W	RPL30	protein biosynthesis
YOR234C	RPL33B	protein biosynthesis
YIL052C	RPL34B	protein biosynthesis
YLR185W	RPL37A	protein biosynthesis
YDR500C	RPL37B	protein biosynthesis
YLR325C	RPL38	protein biosynthesis
YJL189W	RPL39	protein biosynthesis
YKR094C	RPL40B	protein biosynthesis
YHR141C	RPL42B	protein biosynthesis
YPR043W	RPL43A	protein biosynthesis
YPL131W	RPL5	protein biosynthesis
YLR448W	RPL6B	protein biosynthesis
YGL076C	RPL7A	protein biosynthesis
YPL198W	RPL7B	protein biosynthesis
YLL045C	RPL8B	protein biosynthesis
YGL147C	RPL9A	protein biosynthesis
YOL039W	RPP2A	protein biosynthesis
YGR214W	RPS0A	protein biosynthesis
YDR064W	RPS13	protein biosynthesis
YJL191W	RPS14B	protein biosynthesis
YDR447C	RPS17B	protein biosynthesis
YOL121C	RPS19A	protein biosynthesis
YNL302C	RPS19B	protein biosynthesis
YML063W	RPS1B	protein biosynthesis
YGL123W	RPS2	protein biosynthesis
YHL015W	RPS20	protein biosynthesis
YKR057W	RPS21A	protein biosynthesis
YJL136C	RPS21B	protein biosynthesis
YLR367W	RPS22B	protein biosynthesis
YGR118W	RPS23A	protein biosynthesis
YPR132W	RPS23B	protein biosynthesis
YER074W	RPS24A	protein biosynthesis

YIL069C	RPS24B	protein biosynthesis
YGR027C	RPS25A	protein biosynthesis
YLR264W	RPS28B	protein biosynthesis
YLR388W	RPS29A	protein biosynthesis
YNL178W	RPS3	protein biosynthesis
YHR203C	RPS4B	protein biosynthesis
YBR181C	RPS6B	protein biosynthesis
YPL081W	RPS9A	protein biosynthesis
YKR059W	TIF1	protein biosynthesis
YJL138C	TIF2	protein biosynthesis
YML106W	URA5	Not in annotation
YKL056C	YKL056C	biological_process unknown

Known: 65, Unknown: 1, Not in annotation: 1

Group C

YLR175W	CBF5	RNA metabolism
YNL112W	DBP2	RNA metabolism
YPL266W	DIM1	RNA metabolism
YJL125C	GCD14	RNA metabolism
YNL075W	IMP4	RNA metabolism
YDL051W	LHP1	RNA metabolism
YJR002W	MPP10	RNA metabolism
YGL120C	PRP43	RNA metabolism
YGR280C	PXR1	RNA metabolism
YLR197W	SIK1	RNA metabolism
YGR195W	SKI6	RNA metabolism
YLL011W	SOF1	RNA metabolism
YKR056W	TRM2	RNA metabolism
YDR165W	TRM82	RNA metabolism
YLR401C	YLR401C	RNA metabolism
YGL078C	DBP3	RNA metabolism, ribosome biogenesis and assembly
YNR038W	DBP6	RNA metabolism, ribosome biogenesis and assembly
YKR024C	DBP7	RNA metabolism, ribosome biogenesis and assembly
YLR276C	DBP9	RNA metabolism, ribosome biogenesis and assembly
YLR129W	DIP2	RNA metabolism, ribosome biogenesis and assembly
YLL008W	DRS1	RNA metabolism, ribosome biogenesis and assembly
YKL172W	EBP2	RNA metabolism, ribosome biogenesis and assembly
YBR247C	ENP1	RNA metabolism, ribosome biogenesis and assembly
YMR049C	ERB1	RNA metabolism, ribosome biogenesis and assembly
YJL033W	HCA4	RNA metabolism, ribosome biogenesis and assembly
YPR112C	MRD1	RNA metabolism, ribosome biogenesis and assembly
YKL009W	MRT4	RNA metabolism, ribosome biogenesis and assembly
YPL211W	NIP7	RNA metabolism, ribosome biogenesis and assembly
YNL061W	NOP2	RNA metabolism, ribosome biogenesis and assembly
YGR159C	NSR1	RNA metabolism, ribosome biogenesis and assembly
YER006W	NUG1	RNA metabolism, ribosome biogenesis and assembly
YOR145C	PNO1	RNA metabolism, ribosome biogenesis and assembly
YGL171W	ROK1	RNA metabolism, ribosome biogenesis and assembly
YHR088W	RPF1	RNA metabolism, ribosome biogenesis and assembly

YKR081C	RPF2	RNA metabolism, ribosome biogenesis and assembly
YDR087C	RRP1	RNA metabolism, ribosome biogenesis and assembly
YDR083W	RRP8	RNA metabolism, ribosome biogenesis and assembly
YFL002C	SPB4	RNA metabolism, ribosome biogenesis and assembly
YLR435W	TSR2	RNA metabolism, ribosome biogenesis and assembly
YJL109C	UTP10	RNA metabolism, ribosome biogenesis and assembly
YKL099C	UTP11	RNA metabolism, ribosome biogenesis and assembly
YLR222C	UTP13	RNA metabolism, ribosome biogenesis and assembly
YML093W	UTP14	RNA metabolism, ribosome biogenesis and assembly
YMR093W	UTP15	RNA metabolism, ribosome biogenesis and assembly
YJL069C	UTP18	RNA metabolism, ribosome biogenesis and assembly
YDR324C	UTP4	RNA metabolism, ribosome biogenesis and assembly
YDR398W	UTP5	RNA metabolism, ribosome biogenesis and assembly
YDR449C	UTP6	RNA metabolism, ribosome biogenesis and assembly
YGR128C	UTP8	RNA metabolism, ribosome biogenesis and assembly
YHR196W	UTP9	RNA metabolism, ribosome biogenesis and assembly
YDR365C	YDR365C	RNA metabolism, ribosome biogenesis and assembly
YDR412W	YDR412W	RNA metabolism, ribosome biogenesis and assembly
YGR145W	YGR145W	RNA metabolism, ribosome biogenesis and assembly
YGR272C	YGR272C	RNA metabolism, ribosome biogenesis and assembly
YJL010C	YJL010C	RNA metabolism, ribosome biogenesis and assembly
YGR245C	SDA1	cell cycle, cytoskeleton organization and biogenesis
YNL256W	FOL1	coenzyme and prosthetic group metabolism
YGL099W	LSG1	conjugation
YDR312W	SSF2	conjugation
YLR074C	BUD20	cytokinesis
YMR014W	BUD22	cytokinesis
YKL082C	YKL082C	cytoskeleton organization and biogenesis
YBL039C	URA7	lipid metabolism
YIR026C	YVH1	meiosis
YNL062C	GCD10	protein biosynthesis
YMR309C	NIP1	protein biosynthesis
YHR052W	CIC1	protein catabolism
YGR123C	PPT1	protein modification
YDR465C	RMT2	protein modification
YDR101C	ARX1	ribosome biogenesis and assembly
YOL077C	BRX1	ribosome biogenesis and assembly
YGL029W	CGR1	ribosome biogenesis and assembly
YKL078W	DHR2	ribosome biogenesis and assembly
YMR128W	ECM16	ribosome biogenesis and assembly
YLR186W	EMG1	ribosome biogenesis and assembly
YCL059C	KRR1	ribosome biogenesis and assembly
YAL025C	MAK16	ribosome biogenesis and assembly
YHR170W	NMD3	ribosome biogenesis and assembly
YOR206W	NOC2	ribosome biogenesis and assembly
YPR144C	NOC4	ribosome biogenesis and assembly
YPL093W	NOG1	ribosome biogenesis and assembly
YNR053C	NOG2	ribosome biogenesis and assembly
YNL110C	NOP15	ribosome biogenesis and assembly
YER002W	NOP16	ribosome biogenesis and assembly
YGR103W	NOP7	ribosome biogenesis and assembly
YGL111W	NSA1	ribosome biogenesis and assembly

YER126C	NSA2	ribosome biogenesis and assembly
YOL080C	REX4	ribosome biogenesis and assembly
YLR009W	RLP24	ribosome biogenesis and assembly
YMR131C	RRB1	ribosome biogenesis and assembly
YCL054W	SPB1	ribosome biogenesis and assembly
YIR012W	SQT1	ribosome biogenesis and assembly
YDL060W	TSR1	ribosome biogenesis and assembly
YJL148W	RPA34	transcription
YNL248C	RPA49	transcription
YNL113W	RPC19	transcription
YKR025W	RPC37	transcription
YPR110C	RPC40	transcription
YMR269W	YMR269W	Not in annotation
YDR361C	BCP1	biological_process unknown
YLR449W	FPR4	biological_process unknown
YAL036C	FUN11	biological_process unknown
YMR290C	HAS1	biological_process unknown
YGR187C	HGH1	biological_process unknown
YNL132W	KRE33	biological_process unknown
YPL226W	NEW1	biological_process unknown
YNL175C	NOP13	biological_process unknown
YDR496C	PUF6	biological_process unknown
YLR196W	PWP1	biological_process unknown
YBL028C	YBL028C	biological_process unknown
YCR016W	YCR016W	biological_process unknown
YCR072C	YCR072C	biological_process unknown
YDL063C	YDL063C	biological_process unknown
YER036C	YER036C	biological_process unknown
YIL019W	YIL019W	biological_process unknown
YIL127C	YIL127C	biological_process unknown
YJL122W	YJL122W	biological_process unknown
YLR051C	YLR051C	biological_process unknown
YLR221C	YLR221C	biological_process unknown
YNR054C	YNR054C	biological_process unknown
YOL022C	YOL022C	biological_process unknown
YOL124C	YOL124C	biological_process unknown
YPL146C	YPL146C	biological_process unknown
YPR143W	YPR143W	biological_process unknown

Known: 98, Unknown: 25, Not in annotation: 1

**Group D**		
YHR174W	ENO2	carbohydrate metabolism
YKL060C	FBA1	carbohydrate metabolism
YBR196C	PGI1	carbohydrate metabolism
YCR012W	PGK1	carbohydrate metabolism
YJL052W	TDH1	carbohydrate metabolism
YJR009C	TDH2	carbohydrate metabolism
YGR192C	TDH3	carbohydrate metabolism
YDR050C	TPI1	carbohydrate metabolism

YLR044C	PDC1	energy pathways
YLR134W	PDC5	energy pathways

Known: 10, Unknown: 0, Not in annotation: 0

**Group E**		
YGR124W	ASN2	amino acid and derivative metabolism
YCR053W	THR4	amino acid and derivative metabolism
YLR354C	TAL1	carbohydrate metabolism
YLR153C	ACS2	coenzyme and prosthetic group metabolism
YGR204W	ADE3	coenzyme and prosthetic group metabolism
YML126C	ERG13	lipid metabolism
YML008C	ERG6	lipid metabolism
YDR226W	ADK1	Not in annotation

Known: 7, Unknown: 0, Not in annotation: 1

**Group F**		
YMR302C	PRP12	RNA metabolism, organelle organization and biogenesis, ribosome b
YKR048C	NAP1	budding
YMR276W	DSK2	cell cycle
YKL007W	CAP1	cell wall organization and biogenesis
YMR267W	PPA2	cellular respiration
YLR330W	CHS5	conjugation
YKL003C	MRP17	protein biosynthesis
YPR103W	PRE2	protein catabolism
YFR050C	PRE4	protein catabolism
YOL038W	PRE6	protein catabolism
YML092C	PRE8	protein catabolism
YGR135W	PRE9	protein catabolism
YOR157C	PUP1	protein catabolism
YHR200W	RPN10	protein catabolism
YFR004W	RPN11	protein catabolism
YFR052W	RPN12	protein catabolism
YER021W	RPN3	protein catabolism
YDL097C	RPN6	protein catabolism
YPR108W	RPN7	protein catabolism
YKL145W	RPT1	protein catabolism
YDR394W	RPT3	protein catabolism
YOR117W	RPT5	protein catabolism
YGL048C	RPT6	protein catabolism
YGL011C	SCL1	protein catabolism
YJR117W	STE24	protein modification
YER012W	PRE1	response to stress
YJL001W	PRE3	response to stress
YGR253C	PUP2	response to stress
YJL036W	SNX4	transport

YGR048W	UFD1	transport
YGR167W	CLC1	vesicle-mediated transport
YKL002W	DID4	vesicle-mediated transport
YJL053W	PEP8	vesicle-mediated transport
YOR132W	VPS17	vesicle-mediated transport
YOR163W	DDP1	Not in annotation
YKL117W	SBA1	Not in annotation
YKL160W	YKL160W	Not in annotation
YOR042W	CUE5	biological_process unknown
YIR003W	YIR003W	biological_process unknown
YKR016W	YKR016W	biological_process unknown
YPR148C	YPR148C	biological_process unknown

Known: 34, Unknown: 4, Not in annotation: 3

Group G

YNL097C	PHO23	DNA metabolism, nuclear organization and biogenesis
YCR052W	RSC6	DNA metabolism, nuclear organization and biogenesis
YFR037C	RSC8	DNA metabolism, nuclear organization and biogenesis
YGL044C	RNA15	RNA metabolism
YLR298C	YHC1	RNA metabolism
YIL063C	YRB2	nuclear organization and biogenesis
YGL095C	VPS45	organelle organization and biogenesis
YMR005W	TAF4	transcription
YLR025W	SNF7	vesicle-mediated transport
YPR173C	VPS4	vesicle-mediated transport
YBR227C	MCX1	biological_process unknown
YPL105C	YPL105C	biological_process unknown
YPR125W	YPR125W	biological_process unknown
YGL164C	YRB30	biological_process unknown

Known: 10, Unknown: 4, Not in annotation: 0

Group H

YLR377C	FBP1	carbohydrate metabolism
YER065C	ICL1	carbohydrate metabolism
YNL117W	MLS1	carbohydrate metabolism
YKR097W	PCK1	carbohydrate metabolism
YGR070W	ROM1	cell wall organization and biogenesis
YKL217W	JEN1	transport
YJR095W	SFC1	transport

Known: 7, Unknown: 0, Not in annotation: 0

Group I

YKL085W	MDH1	carbohydrate metabolism
YLL041C	SDH2	carbohydrate metabolism
YDR178W	SDH4	carbohydrate metabolism
YGL191W	COX13	cellular respiration
YGL187C	COX4	cellular respiration
YHR051W	COX6	cellular respiration
YLR395C	COX8	cellular respiration
YEL024W	RIP1	cellular respiration
YKL016C	ATP7	coenzyme and prosthetic group metabolism, transport
YJR048W	CYC1	electron transport
YEL039C	CYC7	electron transport
YOR065W	CYT1	electron transport
YML120C	NDI1	electron transport

Known: 13, Unknown: 0, Not in annotation: 0

**Group J**		
YLR304C	ACO1	carbohydrate metabolism
YNL037C	IDH1	carbohydrate metabolism
YOR136W	IDH2	carbohydrate metabolism
YDL066W	IDP1	carbohydrate metabolism
YKL148C	SDH1	carbohydrate metabolism
YCR011C	ADP1	transport
YIL088C	AVT7	transport
YPL265W	DIP5	transport

Known: 8, Unknown: 0, Not in annotation: 0

**Group K**		
YML128C	MSC1	DNA metabolism
YLR270W	DCS1	RNA metabolism
YMR170C	ALD2	amino acid and derivative metabolism
YDR129C	SAC6	budding, response to stress, vesicle-mediated transport
YBR149W	ARA1	carbohydrate metabolism
YCL040W	GLK1	carbohydrate metabolism
YMR105C	PGM2	carbohydrate metabolism
YJR096W	YJR096W	carbohydrate metabolism
YDR368W	YPR1	carbohydrate metabolism
YEL011W	GLC3	carbohydrate metabolism, energy pathways
YMR311C	GLC8	carbohydrate metabolism, energy pathways
YFR015C	GSY1	carbohydrate metabolism, energy pathways
YLR258W	GSY2	carbohydrate metabolism, energy pathways
YEL012W	UBC8	carbohydrate metabolism, protein modification
YBR126C	TPS1	carbohydrate metabolism, response to stress
YDR074W	TPS2	carbohydrate metabolism, response to stress
YKL193C	SDS22	cell cycle
YML004C	GLO1	coenzyme and prosthetic group metabolism
YDR171W	HSP42	cytoskeleton organization and biogenesis, response to stress

YML054C	CYB2	electron transport
YIL124W	AYR1	lipid metabolism
YOR036W	PEP12	organelle organization and biogenesis, transport, vesicle-mediated tr
YGR076C	MRPL25	protein biosynthesis
YER050C	RSM18	protein biosynthesis
YNL007C	SIS1	protein biosynthesis
YKL103C	LAP4	protein catabolism
YLR178C	TFS1	protein catabolism
YJL164C	TPK1	pseudohyphal growth
YDR214W	AHA1	response to stress
YMR250W	GAD1	response to stress
YDL022W	GPD1	response to stress
YCL035C	GRX1	response to stress
YMR186W	HSC82	response to stress
YLL026W	HSP104	response to stress
YPL240C	HSP82	response to stress
YJL034W	KAR2	response to stress
YER103W	SSA4	response to stress
YGR008C	STF2	response to stress
YML100W	TSL1	response to stress
YDR513W	TTR1	response to stress
YCL033C	YCL033C	response to stress
YCL067C	HMLALPHA2	transcription
YCR039C	MATALPHA2	transcription
YGR028W	MSP1	transport
YAL005C	SSA1	transport
YLL024C	SSA2	transport
YGR174C	CBP4	Not in annotation
YLR216C	CPR6	Not in annotation
YOR027W	STI1	Not in annotation
YCR096C	HMRA2	biological_process unknown
YGR248W	SOL4	biological_process unknown
YER175C	TMT1	biological_process unknown
YBR056W	YBR056W	biological_process unknown
YBR280C	YBR280C	biological_process unknown
YCL042W	YCL042W	biological_process unknown
YDL091C	YDL091C	biological_process unknown
YDL124W	YDL124W	biological_process unknown
YER053C	YER053C	biological_process unknown
YHL021C	YHL021C	biological_process unknown
YKL091C	YKL091C	biological_process unknown
YKL151C	YKL151C	biological_process unknown
YLR149C	YLR149C	biological_process unknown
YLR247C	YLR247C	biological_process unknown
YLR251W	YLR251W	biological_process unknown
YLR327C	YLR327C	biological_process unknown
YMR027W	YMR027W	biological_process unknown
YMR110C	YMR110C	biological_process unknown
YNL134C	YNL134C	biological_process unknown
YOL032W	YOL032W	biological_process unknown
YOR215C	YOR215C	biological_process unknown

YPR158W	YPR158W	biological_process unknown

Known: 46, Unknown: 22, Not in annotation: 3

Group L		
YGR256W	GND2	carbohydrate metabolism
YKR076W	ECM4	cell wall organization and biogenesis
YIR038C	GTT1	coenzyme and prosthetic group metabolism
YGR088W	CTT1	response to stress
YIR037W	HYR1	response to stress
YMR322C	SNO4	vitamin metabolism
YOR031W	CRS5	Not in annotation
YDR533C	YDR533C	biological_process unknown
YGR043C	YGR043C	biological_process unknown
YOR391C	YOR391C	biological_process unknown

Known: 6, Unknown: 3, Not in annotation: 1

Group M		
YGR193C	PDX1	coenzyme and prosthetic group metabolism
YCR003W	MRPL32	protein biosynthesis
YGR220C	MRPL9	protein biosynthesis
YOR158W	PET123	protein biosynthesis
YDR175C	RSM24	protein biosynthesis
YNL315C	ATP11	Not in annotation
YDR494W	YDR494W	biological_process unknown

Known: 5, Unknown: 1, Not in annotation: 1

Group N		
YDR263C	DIN7	DNA metabolism
YOR033C	EXO1	DNA metabolism
YML062C	MFT1	DNA metabolism
YLR265C	NEJ1	DNA metabolism
YML065W	ORC1	DNA metabolism
YLL004W	ORC3	DNA metabolism
YFR023W	PES4	DNA metabolism
YIL139C	REV7	DNA metabolism
YPR120C	CLB5	DNA metabolism, cell cycle
YGR109C	CLB6	DNA metabolism, cell cycle
YPL033C	YPL033C	DNA metabolism, meiosis
YPR078C	YPR078C	DNA metabolism, meiosis
YPL178W	CBC2	RNA metabolism
YNR034W	SOL1	RNA metabolism
YIL159W	BNR1	budding, response to stress
YJL155C	FBP26	carbohydrate metabolism

YFL017C	GNA1	carbohydrate metabolism
YDL049C	KNH1	carbohydrate metabolism
YEL061C	CIN8	cell cycle
YER106W	MAM1	cell cycle
YDL028C	MPS1	cell cycle
YLR045C	STU2	cell cycle, cytoskeleton organization and biogenesis
YOR073W	SGO1	cell cycle, meiosis
YDR260C	SWM1	cell cycle, sporulation
YLR220W	CCC1	cell homeostasis
YDR218C	SPR28	cell wall organization and biogenesis
YGR059W	SPR3	cell wall organization and biogenesis
YCR002C	CDC10	cell wall organization and biogenesis, cytokinesis
YLR314C	CDC3	cell wall organization and biogenesis, cytokinesis
YOR212W	STE4	conjugation, signal transduction
YDR103W	STE5	conjugation, signal transduction
YKL189W	HYM1	cytokinesis, morphogenesis
YNL225C	CNM67	cytoskeleton organization and biogenesis
YPL253C	VIK1	cytoskeleton organization and biogenesis
YKL004W	AUR1	lipid metabolism
YGR202C	PCT1	lipid metabolism
YDR273W	DON1	meiosis
YPL130W	SPO19	meiosis
YOL091W	SPO21	meiosis
YBL009W	YBL009W	meiosis
YIL112W	HOS4	meiosis, protein modification
YOR165W	SEY1	membrane organization and biogenesis
YPR140W	YPR140W	membrane organization and biogenesis
YGR099W	TEL2	nuclear organization and biogenesis, response to stress
YLR368W	MDM30	organelle organization and biogenesis
YHR150W	PEX28	organelle organization and biogenesis
YKL167C	MRP49	protein biosynthesis
YKL142W	MRP8	protein biosynthesis
YBR268W	MRPL37	protein biosynthesis
YMR188C	MRPS17	protein biosynthesis
YDR041W	RSM10	protein biosynthesis
YGR185C	TYS1	protein biosynthesis
YLR127C	APC2	protein catabolism
YDR118W	APC4	protein catabolism
YOR249C	APC5	protein catabolism
YHR166C	CDC23	protein catabolism
YFR036W	CDC26	protein catabolism
YBL084C	CDC27	protein catabolism
YLR195C	NMT1	protein modification
YDR523C	SPS1	protein modification
YOR339C	UBC11	protein modification
YER123W	YCK3	protein modification
YJR099W	YUH1	protein modification
YLR227C	ADY4	sporulation
YLR307W	CDA1	sporulation
YLR308W	CDA2	sporulation
YDR403W	DIT1	sporulation
YDR402C	DIT2	sporulation

YER180C	ISC10	sporulation
YIL099W	SGA1	sporulation
YPL027W	SMA1	sporulation
YML066C	SMA2	sporulation
YDR104C	SPO71	sporulation
YGL170C	SPO74	sporulation
YLL005C	SPO75	sporulation
YLR341W	SPO77	sporulation
YOR190W	SPR1	sporulation
YER115C	SPR6	sporulation
YDR522C	SPS2	sporulation
YHR184W	SSP1	sporulation
YOR242C	SSP2	sporulation
YNL128W	TEP1	sporulation
YIR013C	GAT4	transcription
YOL068C	HST1	transcription
YNR007C	ATG3	transport
YBL078C	ATG8	transport
YIR004W	DJP1	transport
YDR508C	GNP1	transport
YNL318C	HXT14	transport
YHR015W	MIP6	transport
YDR113C	PDS1	transport
YGL162W	SUT1	transport
YML097C	VPS9	transport
YLR093C	NYV1	vesicle-mediated transport
YMR017W	SPO20	vesicle-mediated transport
YDR177W	UBC1	vesicle-mediated transport
YOR089C	VPS21	vesicle-mediated transport
YDL080C	THI3	vitamin metabolism
YLR209C	PNP1	Not in annotation
YLR213C	CRR1	biological_process unknown
YDR371W	CTS2	biological_process unknown
YDR516C	EMI2	biological_process unknown
YGR196C	FYV8	biological_process unknown
YOL132W	GAS4	biological_process unknown
YAL018C	YAL018C	biological_process unknown
YBR070C	YBR070C	biological_process unknown
YBR250W	YBR250W	biological_process unknown
YCL048W	YCL048W	biological_process unknown
YDL114W	YDL114W	biological_process unknown
YDR042C	YDR042C	biological_process unknown
YDR065W	YDR065W	biological_process unknown
YDR317W	YDR317W	biological_process unknown
YDR438W	YDR438W	biological_process unknown
YEL023C	YEL023C	biological_process unknown
YEL057C	YEL057C	biological_process unknown
YER085C	YER085C	biological_process unknown
YER182W	YER182W	biological_process unknown
YFL040W	YFL040W	biological_process unknown
YFR032C	YFR032C	biological_process unknown

YGL015C	YGL015C	biological_process unknown
YGL138C	YGL138C	biological_process unknown
YGR226C	YGR226C	biological_process unknown
YGR266W	YGR266W	biological_process unknown
YGR273C	YGR273C	biological_process unknown
YHL012W	YHL012W	biological_process unknown
YIL077C	YIL077C	biological_process unknown
YIL161W	YIL161W	biological_process unknown
YJL037W	YJL037W	biological_process unknown
YJL038C	YJL038C	biological_process unknown
YJL160C	YJL160C	biological_process unknown
YKR005C	YKR005C	biological_process unknown
YKR089C	YKR089C	biological_process unknown
YKR100C	YKR100C	biological_process unknown
YLL012W	YLL012W	biological_process unknown
YLL022C	YLL022C	biological_process unknown
YLR012C	YLR012C	biological_process unknown
YLR030W	YLR030W	biological_process unknown
YLR343W	YLR343W	biological_process unknown
YMR114C	YMR114C	biological_process unknown
YMR144W	YMR144W	biological_process unknown
YNL019C	YNL019C	biological_process unknown
YNL033W	YNL033W	biological_process unknown
YNL034W	YNL034W	biological_process unknown
YOL015W	YOL015W	biological_process unknown
YOL024W	YOL024W	biological_process unknown
YOL047C	YOL047C	biological_process unknown
YOL048C	YOL048C	biological_process unknown
YPR027C	YPR027C	biological_process unknown
YBR148W	YSW1	biological_process unknown

Known: 98, Unknown: 50, Not in annotation: 1

**Group O**		
YHR157W	REC104	DNA metabolism
YHL022C	SPO11	DNA metabolism
YHR014W	SPO13	cell cycle
YOL104C	NDJ1	meiosis
YPR007C	REC8	meiosis
YBR184W	YBR184W	biological_process unknown
YGL081W	YGL081W	biological_process unknown

Known: 5, Unknown: 2, Not in annotation: 0

**Group P**		
YOR074C	CDC21	DNA metabolism
YLR103C	CDC45	DNA metabolism
YPR175W	DPB2	DNA metabolism
YOL090W	MSH2	DNA metabolism
YDR097C	MSH6	DNA metabolism

YBL035C	POL12	DNA metabolism
YNL262W	POL2	DNA metabolism
YBR088C	POL30	DNA metabolism
YJR043C	POL32	DNA metabolism
YKL045W	PRI2	DNA metabolism
YPL153C	RAD53	DNA metabolism
YAR007C	RFA1	DNA metabolism
YJL173C	RFA3	DNA metabolism
YBR087W	RFC5	DNA metabolism
YLR383W	RHC18	DNA metabolism
YIL066C	RNR3	DNA metabolism
YLL002W	RTT109	DNA metabolism
YLR154C	YLR154C	DNA metabolism
YER095W	RAD51	DNA metabolism, nuclear organization and biogenesis, response to s
YAR008W	SEN34	RNA metabolism
YIL140W	AXL2	budding
YGR152C	RSR1	budding
YCL024W	KCC4	budding, cytokinesis, protein modification
YPL256C	CLN2	cell cycle
YDL003W	MCD1	cell cycle
YMR076C	PDS5	cell cycle
YFL008W	SMC1	cell cycle
YNL309W	STB1	cell cycle
YML027W	YOX1	cell cycle
YCR065W	HCM1	cell cycle, transcription
YGL027C	CWH41	cell wall organization and biogenesis
YIL026C	IRR1	conjugation
YNL233W	BNI4	cytokinesis
YJL187C	SWE1	meiosis
YDL101C	DUN1	protein modification
YLR313C	SPH1	pseudohyphal growth
YJL074C	SMC3	sporulation
YOL017W	ESC8	transcription
YMR179W	SPT21	transcription
YPL163C	SVS1	Not in annotation
YGR189C	CRH1	biological_process unknown
YKR013W	PRY2	biological_process unknown
YLR183C	TOS4	biological_process unknown
YGR221C	YGR221C	biological_process unknown
YHR149C	YHR149C	biological_process unknown
YJL181W	YJL181W	biological_process unknown
YKR077W	YKR077W	biological_process unknown
YNL300W	YNL300W	biological_process unknown
YOL007C	YOL007C	biological_process unknown
YOL019W	YOL019W	biological_process unknown
YPL267W	YPL267W	biological_process unknown
YPR174C	YPR174C	biological_process unknown

Known: 39, Unknown: 12, Not in annotation: 1

Group Q		
YER190W	YRF1-2	DNA metabolism, nuclear organization and biogenesis, response to s
YGR296W	YRF1-3	DNA metabolism, nuclear organization and biogenesis, response to s
YLR466W	YRF1-4	DNA metabolism, nuclear organization and biogenesis, response to s
YLR467W	YRF1-5	DNA metabolism, nuclear organization and biogenesis, response to s
YNL339C	YRF1-6	DNA metabolism, nuclear organization and biogenesis, response to s
YBL111C	YBL111C	biological_process unknown
YBL113C	YBL113C	biological_process unknown
YEL075C	YEL075C	biological_process unknown
YEL076C	YEL076C	biological_process unknown
YEL077C	YEL077C	biological_process unknown
YER189W	YER189W	biological_process unknown
YFL064C	YFL064C	biological_process unknown
YFL067W	YFL067W	biological_process unknown
YHL049C	YHL049C	biological_process unknown
YHL050C	YHL050C	biological_process unknown
YHR218W	YHR218W	biological_process unknown
YHR219W	YHR219W	biological_process unknown
YIL177C	YIL177C	biological_process unknown
YJL225C	YJL225C	biological_process unknown
YLL066C	YLL066C	biological_process unknown
YLL067C	YLL067C	biological_process unknown
YLR462W	YLR462W	biological_process unknown
YLR464W	YLR464W	biological_process unknown
YPR202W	YPR202W	biological_process unknown
YPR203W	YPR203W	biological_process unknown
YPR204W	YPR204W	biological_process unknown

Known: 5, Unknown: 21, Not in annotation: 0

Group R		
YNL030W	HHF2	DNA metabolism, nuclear organization and biogenesis
YBR010W	HHT1	DNA metabolism, nuclear organization and biogenesis
YNL031C	HHT2	DNA metabolism, nuclear organization and biogenesis
YDR225W	HTA1	DNA metabolism, nuclear organization and biogenesis
YBL003C	HTA2	DNA metabolism, nuclear organization and biogenesis
YDR224C	HTB1	DNA metabolism, nuclear organization and biogenesis
YBL002W	HTB2	DNA metabolism, nuclear organization and biogenesis

Known: 7, Unknown: 0, Not in annotation: 0

Group S		
YIL129C	TAO3	budding
YJL158C	CIS3	cell wall organization and biogenesis
YBR078W	ECM33	cell wall organization and biogenesis
YIL123W	SIM1	cytoskeleton organization and biogenesis
YJL134W	LCB3	lipid metabolism, signal transduction
YBR243C	ALG7	protein modification

YPL116W	HOS3	protein modification
YAL023C	PMT2	protein modification
YDL145C	COP1	transport, vesicle-mediated transport
YOR247W	SRL1	Not in annotation
YMR215W	GAS3	biological_process unknown
YGL101W	YGL101W	biological_process unknown
YLR455W	YLR455W	biological_process unknown
YMR003W	YMR003W	biological_process unknown

Known: 9, Unknown: 4, Not in annotation: 1

Group T

YLR131C	ACE2	cell cycle
YGL021W	ALK1	cell cycle
YPR119W	CLB2	cell cycle
YAR018C	KIN3	cell cycle
YPL155C	KIP2	cell cycle
YML034W	SRC1	cell cycle
YDR146C	SWI5	cell cycle
YGR108W	CLB1	cell cycle, meiosis
YBR038W	CHS2	cytokinesis
YMR032W	HOF1	cytokinesis
YHR023W	MYO1	cytokinesis
YLR084C	RAX2	cytokinesis
YOR025W	HST3	lipid metabolism
YGL116W	CDC20	protein catabolism
YMR001C	CDC5	protein modification
YIL106W	MOB1	protein modification
YLR190W	MMR1	biological_process unknown
YDR033W	MRH1	biological_process unknown
YIL158W	YIL158W	biological_process unknown
YML119W	YML119W	biological_process unknown
YNL058C	YNL058C	biological_process unknown
YOL070C	YOL070C	biological_process unknown
YPL141C	YPL141C	biological_process unknown
YBR054W	YRO2	biological_process unknown

Known: 16, Unknown: 8, Not in annotation: 0

Group U

YBR158W	AMN1	cell cycle
YNL078W	NIS1	cell cycle
YLR079W	SIC1	cell cycle
YER124C	DSE1	cell wall organization and biogenesis
YHR143W	DSE2	cell wall organization and biogenesis
YJL159W	HSP150	cell wall organization and biogenesis
YKL164C	PIR1	cell wall organization and biogenesis
YKL163W	PIR3	cell wall organization and biogenesis

YGR041W	BUD9	cytokinesis
YLR286C	CTS1	cytokinesis
YNR067C	DSE4	cytokinesis
YNL327W	EGT2	cytokinesis
YGL028C	SCW11	cytokinesis
YIL009W	FAA3	lipid metabolism
YKL185W	ASH1	pseudohyphal growth
YBR083W	TEC1	pseudohyphal growth
YKL116C	PRR1	signal transduction
YGR086C	PIL1	biological_process unknown
YJL078C	PRY3	biological_process unknown
YDR055W	PST1	biological_process unknown
YPL158C	YPL158C	biological_process unknown

Known: 17, Unknown: 4, Not in annotation: 0

Group V

YDR073W	SNF11	DNA metabolism, nuclear organization and biogenesis
YPR134W	MSS18	RNA metabolism
YDR034C	LYS14	amino acid and derivative metabolism
YPR131C	NAT3	protein modification
YJR153W	PGU1	pseudohyphal growth
YER045C	ACA1	transcription
YBR279W	PAF1	transcription
YPR067W	ISA2	transport
YIL167W	YIL167W	Not in annotation
YDR266C	YDR266C	biological_process unknown
YIL165C	YIL165C	biological_process unknown
YJR080C	YJR080C	biological_process unknown

Known: 8, Unknown: 3, Not in annotation: 1

Group W

YJR086W	STE18	conjugation, signal transduction
YJL223C	PAU1	biological_process unknown
YEL049W	PAU2	biological_process unknown
YLR461W	PAU4	biological_process unknown
YFL020C	PAU5	biological_process unknown
YNR076W	PAU6	biological_process unknown
YGR294W	YGR294W	biological_process unknown
YHL046C	YHL046C	biological_process unknown
YIR041W	YIR041W	biological_process unknown
YLL064C	YLL064C	biological_process unknown

Known: 1, Unknown: 9, Not in annotation: 0

Group X		
YKL001C	MET14	amino acid and derivative metabolism
YNL277W	MET2	amino acid and derivative metabolism
YLL062C	MHT1	amino acid and derivative metabolism
YGL184C	STR3	amino acid and derivative metabolism
YKR069W	MET1	amino acid and derivative metabolism, coenzyme and prosthetic grou
YIR017C	MET28	amino acid and derivative metabolism, transcription
YLL061W	MMP1	transport
YGR055W	MUP1	transport
YPL274W	SAM3	transport

Known: 9, Unknown: 0, Not in annotation: 0

Group Y		
YGR089W	NNF2	cell cycle
YOR129C	YOR129C	cell homeostasis
YNL197C	WHI3	morphogenesis
YER114C	BOI2	signal transduction
YKR031C	SPO14	sporulation, vesicle-mediated transport
YJR031C	GEA1	transport, vesicle-mediated transport
YGL139W	YGL139W	biological_process unknown

Known: 6, Unknown: 1, Not in annotation: 0

Group Z		
YGL090W	LIF1	DNA metabolism
YNR044W	AGA1	conjugation
YGL032C	AGA2	conjugation
YJL170C	ASG7	conjugation
YCL027W	FUS1	conjugation
YGL089C	MF(ALPHA)2	conjugation
YIL037C	PRM2	conjugation
YJR004C	SAG1	conjugation
YFL026W	STE2	conjugation
YNL145W	MFA2	conjugation, signal transduction
YKL178C	STE3	conjugation, signal transduction
YCL055W	KAR4	meiosis
YPL192C	PRM3	nuclear organization and biogenesis
YBL016W	FUS3	protein modification
YLR120C	YPS1	protein modification
YLR452C	SST2	signal transduction
YIL079C	AIR1	transport
YNL279W	PRM1	Not in annotation
YPL088W	YPL088W	Not in annotation

Known: 17, Unknown: 0, Not in annotation: 2

Group AA

YNR058W	BIO3	coenzyme and prosthetic group metabolism, vitamin metabolism
YNR056C	BIO5	transport
YPL249C	GYP5	vesicle-mediated transport
YMR077C	VPS20	vesicle-mediated transport
YLR408C	YLR408C	biological_process unknown
YMR073C	YMR073C	biological_process unknown
YMR075W	YMR075W	biological_process unknown
YOL164W	YOL164W	biological_process unknown

Known: 4, Unknown: 4, Not in annotation: 0

Group BB

YKL038W	RGT1	carbohydrate metabolism
YJL047C	RTT101	cell cycle
YDR293C	SSD1	cell wall organization and biogenesis
YKL020C	SPT23	lipid metabolism, response to stress
YDR379W	RGA2	pseudohyphal growth
YDR335W	MSN5	transport
YKL179C	COY1	vesicle-mediated transport

Known: 7, Unknown: 0, Not in annotation: 0

Group CC

YJR010C-A	SPC1	protein modification
YHL048W	COS8	response to stress
YIR043C	YIR043C	Not in annotation
YIR044C	YIR044C	Not in annotation
YBR302C	COS2	biological_process unknown
YML132W	COS3	biological_process unknown
YFL062W	COS4	biological_process unknown
YJR161C	COS5	biological_process unknown
YDL248W	COS7	biological_process unknown

Known: 2, Unknown: 5, Not in annotation: 2

Group DD

YBL019W	APN2	DNA metabolism
YDR076W	RAD55	DNA metabolism
YBR019C	GAL10	carbohydrate metabolism
YBR076W	ECM8	cell wall organization and biogenesis
YGL178W	MPT5	cell wall organization and biogenesis

YAR019C	CDC15	cytokinesis
YJR108W	ABM1	cytoskeleton organization and biogenesis
YCR068W	ATG15	membrane organization and biogenesis
YAL010C	MDM10	organelle organization and biogenesis
YNR002C	FUN34	transport
YDL244W	THI13	vitamin metabolism
YPL276W	FDH2	Not in annotation
YAL031C	FUN21	biological_process unknown
YMR156C	TPP1	biological_process unknown
YAR023C	YAR023C	biological_process unknown
YER066W	YER066W	biological_process unknown
YER185W	YER185W	biological_process unknown
YER187W	YER187W	biological_process unknown

Known: 11, Unknown: 6, Not in annotation: 1

**Group EE**		
YIL153W	RRD1	DNA metabolism, response to stress
YHR044C	DOG1	carbohydrate metabolism
YGR292W	MAL12	carbohydrate metabolism
YNL289W	PCL1	cell cycle
YKL086W	YKL086W	response to stress
YFL050C	ALR2	transport
YNL333W	SNZ2	vitamin metabolism
YFL059W	SNZ3	vitamin metabolism
YGR087C	PDC6	Not in annotation
YNR069C	BSC5	biological_process unknown
YFL052W	YFL052W	biological_process unknown

Known: 8, Unknown: 2, Not in annotation: 1

**Group FF**		
YAR010C	YAR010C	Not in annotation (Ty element transposition)
YBL005W-A	YBL005W-A	Not in annotation (Ty element transposition)
YJR026W	YJR026W	Not in annotation (Ty element transposition)
YJR028W	YJR028W	Not in annotation (Ty element transposition)
YML040W	YML040W	Not in annotation (Ty element transposition)
YMR046C	YMR046C	Not in annotation (Ty element transposition)
YMR051C	YMR051C	Not in annotation (Ty element transposition)

Known: 0, Unknown: 0, Not in annotation: 7
